# Abnormal accumulation of extracellular vesicles in hippocampal dystrophic axons and regulation by the primary cilia in Alzheimer’s disease

**DOI:** 10.1186/s40478-023-01637-3

**Published:** 2023-09-04

**Authors:** Jaemyung Jang, Seungeun Yeo, Soonbong Baek, Hyun Jin Jung, Mi Suk Lee, Seung Hee Choi, Youngshik Choe

**Affiliations:** 1 Korea Brain Research Institute, Daegu, 41068 Korea; 2 Daegu, Korea

**Keywords:** Dystrophic neurites, Primary cilia, Hippocampal septal connection, Extracellular vesicles, Single cell RNA sequencing, Selectively vulnerable neurons

## Abstract

Dystrophic neurites (DNs) are abnormal axons and dendrites that are swollen or deformed in various neuropathological conditions. In Alzheimer’s disease (AD), DNs play a crucial role in impairing neuronal communication and function, and they may also contribute to the accumulation and spread of amyloid beta (Aβ) in the brain of AD patients. However, it is still a challenge to understand the DNs of specific neurons that are vulnerable to Aβ in the pathogenesis of AD. To shed light on the development of radiating DNs, we examined enriched dystrophic hippocampal axons in a mouse model of AD using a three-dimensional rendering of projecting neurons. We employed the anterograde spread of adeno-associated virus (AAV)1 and conducted proteomic analysis of synaptic compartments obtained from hippocampo-septal regions. Our findings revealed that DNs were formed due to synaptic loss at the axon terminals caused by the accumulation of extracellular vesicle (EV). Abnormal EV-mediated transport and exocytosis were identified in association with primary cilia, indicating their involvement in the accumulation of EVs at presynaptic terminals. To further address the regulation of DNs by primary cilia, we conducted knockdown of the *Ift88* gene in hippocampal neurons, which impaired EV-mediated secretion of Aβ and promoted accumulation of axonal spheroids. Using single-cell RNA sequencing, we identified the septal projecting hippocampal somatostatin neurons (SOM) as selectively vulnerable to Aβ with primary cilia dysfunction and vesicle accumulation. Our study suggests that DNs in AD are initiated by the ectopic accumulation of EVs at the neuronal axon terminals, which is affected by neuronal primary cilia.

**Graphical abstract:**

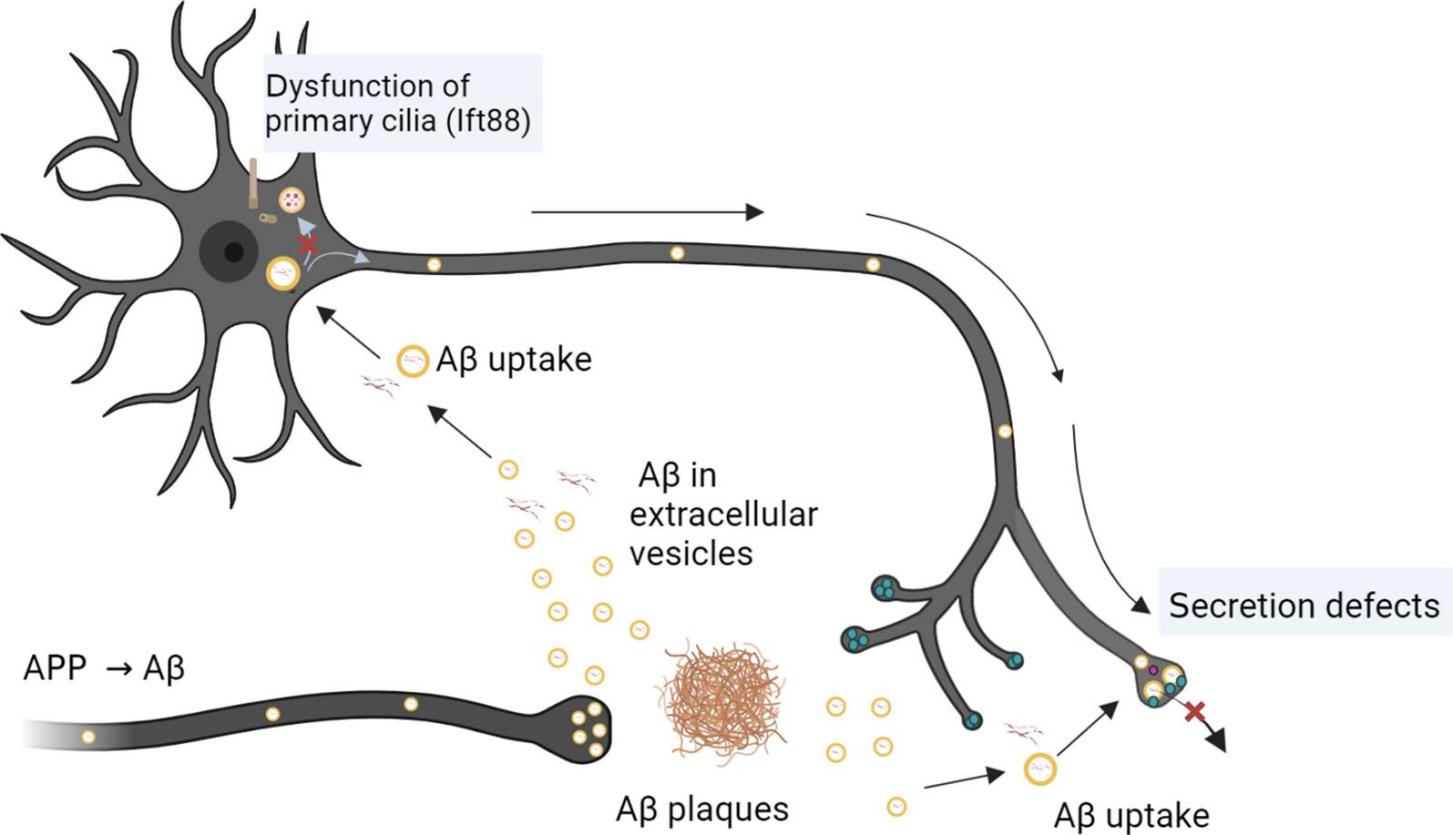

**Supplementary Information:**

The online version contains supplementary material available at 10.1186/s40478-023-01637-3.

## Introduction

The gradual accumulation of amyloid-β (Aβ) begins in the early stages of Alzheimer’s disease (AD) and initiates a series of events resulting in synaptic loss, neuroinflammation, the formation of neurofibrillary tangles, and neuronal dysfunction [27, 30, 71]. The Aβ monomers come together into small soluble oligomers, which are toxic to neurons. These oligomers then change into protofibrils, and subsequently evolve into insoluble fibrils or other transformed structures over time. The formation and aggregation of Aβ fibrils is central to extracellular Aβ plaques, which are accompanied by dystrophic neurites (DNs) comprising swollen axons and terminals [64]. The DNs, termed amyloid-associated axonal spheroids, are globular in shape and have pathological structures that contain cytoskeletal elements, pathological proteins, and damaged organelles, in addition to enzymes involved in amyloid precursor protein (APP) processing [13, 64, 68, 82]. The appearance of DNs is associated with problems with synaptic function, axonal transport, and inflammatory and oxidative stress pathways, making them the first sign of axonal damage before synaptic failure occurs in the early stages of AD [66]. However, it remains incompletely understood how they form along the axon in the context of Aβ accumulation and impair neuronal function.

The Aβ aggregation usually starts in the presynaptic axon terminals, where APP is transported by vesicles and becomes enriched [33, 42, 53]. However, not all neurons are susceptible to the intraneuronal accumulation of Aβ by expressing high levels of the human APP gene and producing Aβ deposits at their axon terminals. Neuronal subtypes that are known to be lost in early AD consist of large pyramidal neurons in layer II of the entorhinal cortex (EC) [29, 70], the CA1 region of the hippocampus [63], and cholinergic [16] and adrenergic [4] neurons in other brain regions. Several studies support the specific neuronal subtypes in the EC that are vulnerable to AD [23, 45], but the neuronal subtypes with the swollen DNs remain undefined. It is not known which types of neurons are selectively susceptible to Aβ aggregation and associated with the development of swelling DNs in the neural circuits of the hippocampal areas. Defective endosome-lysosome-exosome pathways result in Aβ accumulation, atrophy, and damage of the forebrain cholinergic neurons [54]. The movement of vesicles and endo-lysosomal compartments along axons is a key part of amyloid fibril formation and vesicle-mediated aggregation of Tau proteins [7, 87]. Axonal swelling, or spheroid, prominent in neuritic plaques, is formed by abnormal vesicular components in the axons [66, 68]. Therefore, neurons with long-range projecting axons may be more susceptible to accumulating abnormalities in axonal vesicle trafficking associated with age or neurodegenerative disease. However, it has not been possible to characterize vulnerable neuronal types bearing DNs with three-dimensional axonal projection patterns.

We employed various methods to find out how vesicle accumulation in axons contributes to neurite dystrophy in the AD mouse brain. These methods included clearing the aged brain for three-dimensional imaging of DNs, proteomic analysis of defective synaptic compartments, and single-cell RNA sequencing (scRNAseq) to characterize the vulnerable neurons with DNs. In the presence of Aβ, neurons exhibited significant alterations, including axonal enlargement, the accumulation of EVs at axon terminals, and the impairment of primary cilia. These pathological changes at the cellular and molecular level in the AD mouse brain were further supported by conditional knockout of the Ift88 gene in vivo and in vitro. The characterization of neurons with vulnerable axons will shed light on the exact picture of dystrophic axon development and synapse loss along Aβ plaque accumulation in the early stages of AD progression.

## Materials and methods

### Animals

All experimental procedures on animals were conducted according to the Korea Brain Research Institute ethical guidelines and approved by Korea Brain Research Institute Ethical Committee for Animal Experimentation (approved protocol numbers IACUC-19-00014). Mice were kept in ventilated cages under standard housing conditions with ad libitum access to food and water on a standard 12 h light/ 12 h dark cycle.

5xFAD (also known as Tg6799, Jax. #34840, B6SJL-Tg(APPSwFlLon,PSEN1*M146L*L286V)6799Vas/Mmjax) [55] and Thy1-YFP (Jax. #003782, *B6.Cg-Tg(Thy1-YFP)HJrs/J*) [19] mice were purchased from the Jackson Laboratory and C57BL/6 mice via Orient Bio. (South Korea). To visualize the axonal projection of pyramidal neurons, heterozygous male 5xFAD mice were crossed with female Thy1-YFP mice to obtain double transgenic mice (5xFAD; Thy1-YFP). The 5xFAD; Thy1-YFP mice were backcrossed to C57BL/6 mice and maintained in a hemizygous state.

Ai6 mice with Cre-dependent expression of ZsGreen (Jax. #007906, *B6.Cg-Gt(ROSA)26Sortm6(CAG-ZsGreen1)Hze/J*) were purchased from the Jackson Laboratory via Central Lab. Animal Inc. (South Korea). Adult male 5xFAD mice and their corresponding Ai6 littermates were used for the experiments. 5xFAD; Ai6 mice were backcrossed to C57BL/6 mice and maintained in a hemizygous state.

Ift88-flox/flox (Jax. #022409, *B6.129P2-Ift88tm1Bky/J*) were purchased from the Jackson Laboratory via Central Lab. Animal Inc. (South Korea). Adult male 5xFAD and their corresponding Ift88-flox/flox littermates were used for the experiments. 5xFAD; Ift88-flox/flox mice and 5xFAD; Ift88-flox/wt mice were used to examine the effect of the loss of function of the Ift88 allele in a 5xFAD background.

### AAV vector construction

A recombinant AAV vector was created by combining three different plasmids: pAAV-CAG-ArchT-tdTomato (#29778, Addgene plasmid, a gift from Edward Boyden), pAAV-GFP/Cre (#49056, Addgene plasmid, a gift from Fred Gage), and CD63-pEGFP C2 (#62964, Addgene plasmid, a gift from Paul Luzio). The pAAV-CAG-CD63-GFP plasmid was generated by inserting a human tetraspanin CD63 CD63 (*BamHI* and *XhoI* fragment) and EGFP (*XhoI*-*HindIII* fragment) into the pAAV-CAG-ArchT-tdTomato plasmid (*BamHI*-*HindIII* fragment), with the removal of ArchT-tdTomato using *BamHI* and *HindIII*. The resulting plasmid, pAAV-CAG-CD63-GFP, was propagated in Stbl3 *E. coli* cells (Invitrogen™ One Shot™ Stbl3™ Chemically Competent *E. coli*, C737303, ThermoFisher Scientific, USA) and sequenced (Bionics, South Korea) to check for correct expression.

### Virus production and purification

AAV1 and AAV-DJ/8 (Cell Biolabs, Inc., USA) were used with pHelper (Takara, Japan) to produce AAV-GFP/Cre, and AAV-CAG-CD63-EGFP, respectively in HEK293 cells (VirusExpress® 293 AAV Production Cells, VP002, ThermoFisher Scientific, USA) using Polyethylenimine [21]. The cells were transfected with the plasmids and incubated for 72 h. After incubation, the culture medium was collected and centrifuged at 2500 g for 30 min at 4 degrees Celsius (°C) to remove the cell debris. The supernatant was precipitated with 40% w/v PEG-8000 in 0.4 M NaCl overnight at 4 °C. The precipitate was resuspended with phosphate buffered saline (PBS, 10,010,023, Gibco™, USA) 200 mM NaCl, 0.001% Pluronic F68 (ThermoFisher Scientific, USA) and incubated with 50 units/mL of Benzonase® Nuclease (Sigma-Aldrich, USA) at 37 °C for 30 min to lyse any DNA. The lysate was sonicated 4 times at one-second-interval pulses for at least 15 min on ice to break up any large particles. The supernatant was centrifuged at 3220×*g* for 15 min to remove any debris. The clear supernatant was transferred to a tube containing the resuspended virus collected from the culture medium. The total crude virus was purified over iodixanol (OptiPrep™ Density Gradient Medium, D1556, Sigma-Aldrich, USA) density gradients [88] (15%, 25%, 40% and 60%). The recovered AAVs were concentrated, and the buffer was exchanged with virus buffer using Amicon 100 K columns (USC9100, Merck Millipore, USA). The number of genome-containing particles in AAV preparations was determined using a quantitative polymerase chain reaction.

### Stereotaxic injections

For stereotaxic surgery, mice were anesthetized with Avertin (150 mg/kg, 2,2,2-tribromoethanol, Sigma-Aldrich, USA) and mounted on a stereotaxic system. Erythromycin ophthalmic ointment was put on the cornea to keep it from drying out, and a heating pad was used to keep the body temperature at 37 °C. A small craniotomy hole was created using a dental drill, and injections were administered using a micropipette coupled to a nanoliter injector and its controller at a slow flow rate of 0.1 µl/minute. After injecting the virus, we applied the suture clips to the incision and put the mice under a red light to heal the wounds. Post-operative care included buprenorphine injections as required by the Korea Brain Research Institute Ethical Committee for Animal Experimentation. Brain tissues were collected 14 days after AAV injection with perfusion in 4% paraformaldehyde for immunostaining and three-dimensional imaging.

AAV-hSyn-EGFP (#50465-AAV5, Addgene, USA) was injected into the somatosensory cortex layer 2/3 (coordinates: A/P − 0.6 mm, M/L ± 3.2 mm, D/V − 0.3 mm). AAV- GFP/Cre and AAV-CAG-CD63-EGFP were injected into the ventral dentate gyrus (coordinates: A/P 0.4 mm, M/L ± 2.9 mm, D/V − 2.7 mm). AAV1-DJ-GFP was injected into the ventral hippocampus (coordinates: A/P − 2.9 mm, M/L ± 2.5 mm, D/V − 4.0 mm).

### Primary hippocampal neuron culture for transfection

Prenatal Hsd: ICR (CD-1^®^) mice at a gestational age of 14 days were obtained from Koatech (Korea). All animals were maintained under specific pathogen-free conditions, and all research was conducted in accordance with the ethical guidelines of the Korea Brain Research Institute. The cerebral hemispheres were removed, and the hippocampi were separated from the meninges. The hippocampal tissues were gently washed with Hanks’ Balanced Salt Solution Buffer (14025092, Gibco™, USA) containing 10 mM HEPES (15630106, Gibco™, USA), 0.002% deoxyribonuclease I (DN25, Sigma-Aldrich, USA), and 0.025% trypsin (Sigma-Aldrich, USA) at 37 °C for 20 min. This was followed by mechanical trituration using a Pasteur pipette. Following the introduction of DMEM/F-12 (11320033, Gibco™, USA) containing 10 mM HEPES, 1% penicillin/streptomycin (15140122, Gibco™, USA), and 10% FBS (Hyclone, USA), single cells were collected by passing through a 70 μm nylon cell strainer (SPL, Korea) by removing cell clumps. Poly-D-lysine (P6407, Sigma-Aldrich, USA) coated plates were used to seed primary neurons, which were subsequently cultured in DMEM/F-12 supplemented with 10 mM HEPES, 1% penicillin/streptomycin and 10% FBS (Hyclone, USA) at 37 °C with 5% CO2. After 120 min, the medium was replaced with neurobasal medium (21103049, Gibco™, USA), which consisted of 10 mM HEPES, 1% penicillin/streptomycin and a B27 supplement (17504044, Gibco™, USA).

### Transfection

SiRNA transfection was performed with Lipofectamin (Lipofectamine RNAiMAX Transfection Reagent, ThermoFisher Scientific, USA). Primary cultured neurons were transfected with 40 nM of mouse siIft88 or negative control siRNA (Bioneer, Korea). The control siRNA (AccuTarget™ Negative Control siRNA, Bioneer, South Korea) was used and its sequence information was not provided. The sequence of siRNA is as follows: mouse siIft88 siRNA (ACUGGGAGAGUUAUACGAU).

### Preparation and treatment of Aβ1-42

The preparation procedure for Aβ 1-42 (Bachem AG, Switzerland) involved the incubation of fresh solubilized peptides at a concentration of 500 μM in sterile distilled water for a period of 5 days at a temperature of 37 °C [60]. Primary hippocampal neurons were treated with 1 μM Aβ and distilled water as a vehicle at 6 h after transfection and harvested after 42 h.

### Brain fixation and polymerization

To anesthetize the mice, we employed the identical approach described in line 136. Avertin (150 mg/kg, 2,2,2-tribromoethanol, Sigma-Aldrich) was used to anesthetize mice. Mice were infused with ice-cold PBS followed by a cold 4% paraformaldehyde solution. The cerebral hemispheres were quickly removed, immersed in the same paraformaldehyde solution, and postfixed for 4 h at 4 °C in the dark. After fixation, the brains were washed three times using PBS.

To polymerize the fixed brains, a hydrogel solution was employed. The hydrogel solution contains four essential chemicals: acrylamide, bis-acrylamide, formaldehyde, and a thermal initiator (VA-044, Wako Chemicals USA Inc., USA). The brains were transferred into the hydrogel solution and placed in a sealed vacuum oven at 50 °C for 12 h to remove air bubbles and catalyze the polymerization process. After the hydrogel had polymerized, excess gel and the remaining hydrogel solution were washed by placing the brains into a 50-mL tube containing new PBS.

### Tissue clearing using active tissue clearing technique

We modified ACT-PESTRO as previously reported [43]. To remove excess lipid contents from 5xFAD; Thy1-YFP mouse brains, we used X-Clarity tissue clearing system II (Logos Biosystems, Korea) that was equipped with platinum plates for high-density current. The brains were polymerized as described above and cleared for 24 h in the X-Clarity system for 24 h. Then, samples were incubated at 37 °C for 1–2 days with primary antibodies (1:200) in 0.1% tween 20 in PBS (PBST). After washing with PBST, tissues were incubated at 37 °C for 1–2 days with secondary antibodies conjugated with Alexa Fluor 488, 568 (1:400, Invitrogen, USA). To match refractive index before imaging, cleared brain tissues were immersed in RI matching solution (50% sucrose (Sigma-Aldrich, USA)/25% urea (Sigma-Aldrich, USA) for 24–48 h. For imaging Aβ plaques in the cleared mouse brain, we used an anti-Aβ antibody (D54D2, Cell Signaling Technology, USA) by treating the cleared brain tissues using the DeepLabel Antibody Staining Kit (Logos Biosystems, Korea).

The sample was imaged using a light-sheet fluorescence microscope (Ultramicroscope II, LaVision BioTec GmbH, Germany) with dipping cap, an NKT Photonics SuperK EXTREME EXW-12 white light laser, an Andor Neo sCMOS camera, and a homemade sample holder. The numerical size of the light sheet was set to 0.73 and optical zoom was set to 0.65. For GFP and Aβ imaging, the excitation filters were 470/40 and 560/25, and the emission filters were 525/50 and 620/60, respectively. The step size of the scan was set to 3 μm, and each channel was taken in two separate scans. The serial TIFF image files were converted to the Imaris file format so that Imaris software (Bitplane, Cologne, Germany) could be used to perform post-processing and three-dimensional rendering of images.

### Immunohistochemistry

For immunohistochemical analysis, the tissue sections were treated with 1 × tris-buffered saline (TBS), permeabilized with 1 × TBS-T (1 × TBS with 0.05% Tween20), and nonspecific binding of antibodies was eliminated using blocking buffer (2% heat-inactivated normal goat serum, 2% bovine serum albumin in 1 × TBS-T) for 1 h at room temperature. The sections were incubated overnight at 4 °C with primary antibodies at predetermined dilutions in blocking buffer, followed by incubation with a secondary antibody in blocking buffer for 30 min at room temperature. Images were acquired using an upright confocal microscope (C1, Nikon, Japan), two inverted confocal microscopes (A1/NiE Nikon, Japan or TCS SP8, Leica, Germany). All representative images were presented (n = 4, female = 4), unless otherwise noted. The primary antibodies used for immunostaining were listed below and used at a concentration of 1:300–1:1000. Alexa Fluor-conjugated secondary antibodies raised from goat (ThermoFisher Scientific, USA) were used.

### Antibody and dye list

**Table:** 

Target	Antibody or dye	Company	Cat. No	Clonality
Amylo-Glo	Amylo-Glo RTD Amyloid Plaque Stain Reagent	Biosensis	TR-300-AG	N.A
AC3	AC3	Abcam	ab125093	Rabbit
Ctip2	Ctip2 [25B6]	Abcam	ab18465	Rat
Lrp1	LRP1	Abcam	ab92544	Rabbit
Map2	MAP2	Abcam	ab5392	Chicken
MBP	MBP	AvesLabs	MBP	Chicken
pSer8-Aβ	Phospho Amyloid-β, Ser8 [1E4E11]	Kerafast	EBN001	Mouse
vGlut1	VGluT1	Abcam	ab104898	Rabbit
CD63	CD63	Sicgen	AB0047-200	Goat
DAPI	DAPI (4′,6-Diamidino-2-Phenylindole, Dilactate)	Life Technologies	D3571	N.A
Hoechst	Hoechst 33,342 Solution (20 mM)	ThermoFisher Scientific™	62,249	N.A
IBA1	IBA 1 (AIF 1)	SYSY	234,003	Rabbit
Ift88	IFT88	ABclonal	A4204	Rabbit
MIF	MIF	Bioss	bs-1044R	Rabbit
Tau (AT8)	Phospho-PHF-tau pSer202 + Thr205 (AT8)	ThermoFisher Scientific™	MN1020	Mouse
pSIVA Apoptosis Detection Kit	pSIVA Apoptosis Detection Kit	Novus	NBP2-29,382	N.A
NF	Purified anti-Neurofilament Marker	BioLegend	837,904	Mammalian
Shank2	Shank2	Neuromab	75–088	Mouse
SST	SST	Ptglab	20,404-1-AP	Rabbit
Tau	Tau (D1M9X) XP^®^	Cell Signaling	46,687	Rabbit
Ubiquitin	Ubiquitin (P4D1)	Cell Signaling	3936S	Mouse
6E10	β-amyloid (1–16) (6E10)	Biolegend	803,001	Mouse
D54D2	β-amyloid (D54D2) XP®	Cell Signaling	8243S	Rabbit

### Preparation of lysate and synaptosomes

The cortex, hippocampus, and septum of whole mouse brains were dissected and washed with 2 mL of ice-cold D-PBS (14040117, Gibco™, USA). The tissues were then homogenized in 8 M Urea Lysis Buffer (8 M Urea, 2% SDS, 5 mM TEAB, and 150 mM NaCl) and protease inhibitors (Halt™ Protease and Phosphatase Inhibitor Cocktail, 87,786, ThermoFisher Scientific, USA).

The proteins in the lysate were solubilized by incubation on ice for 30 min with shaking. To achieve complete solubilization, the tissue homogenate was sonicated three times at 5-s interval pulses at 20% amplitude using a microtip probe. To obtain insoluble protein fractions, total brain homogenates were centrifuged at 12,000 rpm for 10 min at 4 °C. The supernatant was retained as SDS-soluble protein fractions for western blot analysis.

For the isolation of synaptosomes containing functional synaptic proteins from mouse brain tissues, the brains were rapidly dissected, and the cortex, the hippocampus, and the hippocampo-septal tracts were homogenized in Syn-PER solution (87793, ThermoFisher Scientific, USA) to a final concentration of 100 mg/ml. All steps were performed at 4 °C or on ice, and all buffers were supplemented with protease inhibitors (Halt™ Protease and Phosphatase Inhibitor Cocktail, 87786, ThermoFisher Scientific, USA). The samples were centrifuged at 1200 g for 10 min. The supernatant was centrifuged at 15,000 g for 20 min to isolate synaptosome. Prior to further processing, the synaptosome was resuspended in 8 M Urea Lysis Buffer (8 M Urea, 2% SDS, 5 mM TEAB, and 150 mM NaCl) and protease inhibitors (Halt™ Protease and Phosphatase Inhibitor Cocktail, 87786, ThermoFisher Scientific, USA).

## Western blot

The total protein concentration of the samples obtained by the above procedure was measured using the BCA protein assay kit (23,227, ThermoFisher Scientific, USA), and 15 μg protein was loaded onto a precast polyacrylamide gradient gel (4–20% Mini-PROTEAN^®^ TGX™ Precast Protein Gels, Bio-Rad Laboratories, Inc., USA). Proteins were transferred to polyvinylidene fluoride membranes (Immobilon^®^-P PVDF Membrane, IPVH0010, Millipore, USA). Membranes were blocked for 30 min in 5% skim milk (232100, BD Biosciences, USA) in 1 × PBS-T containing 0.1% Tween 20 (Merck Millipore, USA) and incubated with primary antibodies at 4 °C overnight. After washing, the HRP-conjugated secondary antibodies were incubated for 30 min at room temperature. HRP signals were visualized using an Enhanced Chemiluminescence Reagent Kit (WP20005, ThermoFisher Scientific, USA).

**Table:** 

Target	Antibody	Company	Cat. No	Clonality
CD9	BD Pharmingen™ Purified Mouse Anti-Human CD9	BD Biosciences	555370	Mouse
CD63	BD Pharmingen™ Purified Mouse Anti-Human CD63	BD Biosciences	556019	Mouse
CD81	BD Pharmingen™ Purified Mouse Anti-Human CD81	BD Biosciences	555675	Mouse
Synapsin1	Synapsin1	SYSY	106011	Mouse
Synaptophysin	Synaptophysin (YE269)	Abcam	ab32127	Rabbit
PSD95	PSD95 (D27E11) XP^®^	Cell Signaling	3450S	Rabbit
β-Actin	HRP-Conjugated Beta Actin Antibody	Proteintech	HRP-60008	Mouse
Aβ	β-amyloid (D54D2) XP^®^	Cell Signaling	8243S	Rabbit

### Co-immunoprecipitation (co-IP)

Cells were lysed in a buffer containing 50 mM Tris–Cl pH 7.4, 150 mM NaCl, 1 mM EDTA, 1% Triton X-100, and a proteinase inhibitor. Antibodies were subjected to incubation with Dynabeads M-280 sheep anti-rabbit IgG (Life Technology, USA) in accordance with the instructions provided by the manufacturer. The lysates were subjected to an overnight incubation at 4 °C with the bead-antibody complex. After repeated washes with PBST, the beads were combined with sample buffer, which was adjusted to final 4% SDS by adding SDS (Sigma-Aldrich, 75746, USA) and boiled for 10 min at 100 °C to elute APP-binding proteins. To demonstrate co-immunoprecipitation against APP, the eluted proteins were separated in an SDS-PAGE gel.

To perform co-IP in synaptosomal proteins, 3 µg anti-APP (Abcam, USA) and CD63 (Sicgen, USA) antibodies were incubated with 0.5 mg/ml synaptosomal lysates in a total volume of 0.5 ml at 4 °C in rotating tubes overnight. The antibody-synaptosomal lysate mixture was incubated with 20 µl Dynabeads™ Protein G (Invitrogen, USA) at 4 °C in rotating tubes for 2 h. After incubation, Dynabeads were washed with Syn-PER™ reagent. The beads were eluted in 30 μl 8 M Urea lysis buffer (8 M Urea, 2% SDS, 5 mM TEAB, and 150 mM NaCl) and protease inhibitors (Halt™ Protease and Phosphatase Inhibitor Cocktail, 87786, ThermoFisher Scientific, USA).

### MS analysis

Protein samples were reduced with 2 μl of 500 mM DL-dithiothreitol for 30 min at 55 °C. The samples were alkylated with 4 μl of 500 mM iodoacetamide for 20 min at room temperature in the dark. Trypsin digestion was performed overnight at 37 °C with an enzyme/substrate ratio of 1:50. For label-free proteomics, samples were purified from any contaminants using a Pierce™ C18 spin column (ThermoFisher Scientific, USA), vacuum dried, and stored at -20 °C until further use. Dried peptides from each sample were resuspended in 20 μl of 0.1% formic acid and analyzed using a nano-liquid chromatography system (UltiMate 3000, ThermoFisher Scientific, USA) coupled to a Q-Exactive Plus Orbitrap mass spectrometer (ThermoFisher Scientific, USA). A binary solvent system consisting of 0.1% formic acid in water and 0.1% formic acid in acetonitrile was used for all analyses. Peptide fractions were separated on an Ultimate 3000 RSLC nano system (ThermoFisher Scientific, USA) using a PepMap 100 C18 LC column (#164535, ThermoFisher Scientific, USA) as a loading column, followed by a PepMap RSLC C18 (#ES903, ThermoFisher Scientific, USA) analytical column at a flow rate of 0.3 μl/min for 135 min. Full scan MS with data-dependent MS/MS acquisition was performed in the range of 350–2000 m/z.

### Proteomic analysis

Proteome Discoverer™ (PD; version 2.4; ThermoFisher Scientific, USA) software was used for protein identification and quantification. Raw MS data were searched against a protein database obtained from the UniProt Knowledgebase Release 2022_01 (23-Feb-2022). Searches are performed using the standard workflow with SEQUEST search algorithms as provided in PD.

Data exported from the PD software were transferred to the R environment and analyzed following the analysis workflow in the Differential Enrichment Analysis of Proteomics Data [86]. The abundance values of identified proteins were log-transformed and normalized according to the algorithms of PD. A comparison of differentially expressed proteins between two types of experimental groups is shown in Venn diagrams [20]. Differential enrichment analysis was performed based on the algorithms of PD. An adjusted *p*-value less than 0.1 and an enrichment threshold greater than 0.25 were used as cut-off criteria. The results of the differential enrichment analysis were plotted as volcano plots using the R package EnhancedVolcano [35] or as scatter plots using the R package ggplot2 [73]. And they were delivered to the R package Enrichr [40, 79] and processed by enrichment analysis using the biological process of GO. Cytoscape (version 3.9.1) [67] was used to generate a network of these proteins, mapping protein data onto a PPI network. The network was constructed using the STRING database program [17] available in the public database area of Cytoscape.

### Preparation of microfluidic device

The microfluidic device was designed using AutoCAD(Autodesk Inc., USA), and chrome masks were made (microFIT Co. Ltd, Korea). The SU-8 mold was fabricated using SU8-5 and SU8-100 resin (MicroChem, USA) onto a silicon wafer according to the manufacturer’s instructions in the cleanrooms at the DGIST Institute of Next-generation Semiconductor Convergence Technology. Next, amino groups were generated on SU8 mold via chemical vapor deposition of (3-aminopropyl)triethoxysilane (APTS, Sigma Aldrich, USA) for overnight.

Polydimethylsiloxane (PDMS) mixtures (Sylgard 184, Dow Corning Midland, USA) with pre-polymer: curing agent ratios of 10:1 were poured on the SU-8 mold. They were cured for 1 h at 95 °C on a hot plate and peeled off from the mold. Before being used for culture, the devices were washed with a 70% ethanol solution and autoclaved water, and UV-sterilized for 1 h. The surface of a PDMS replica and a coated glass substrate were exposed to reactive oxygen plasma using a plasma cleaner (CUTE, Femtoscience, Korea) for 1 min to generate hydroxyl functionalities. Finally, they were visually joined to form an irreversible seal, and the PDMS channels were treated with 5 mg/mL poly-D-lysine (Sigma Aldrich, USA) overnight in a humidified incubator at 37 °C (5% CO_2_).

### Primary neuron culture in a microfluidic device

Primary neuron cultures were prepared from 5 and 5xFAD; Ift88-flox/flox mouse pups as previously described. Briefly, two hemispheres were removed from mouse pups, and the hippocampus was separated from the brain and dissociated with trypsin for 15 min at 37 °C. Dissociated cells were seeded in a microfluidic channel at a density of approximately 6 × 10^6^ cells/ml. The neuronal cells were cultured in the neurobasal medium™ supplemented with 2% supplement B27 ™, 0.25% GlutaMAX™ supplement (35050061, Gibco, USA), and 100 µg/ml Primocin^®^ (InvivoGen, Hong Kong) at 37 °C in a humidified atmosphere with 5% CO_2_. The media were replaced with fresh media containing B27™, GlutaMAX™, and 1 µM cytosine β-D-arabinofuranoside hydrochloride (C6645, Sigma-Aldrich) two days after plating.

### Generation of single-cell suspension for scRNAseq analysis

The whole procedure of single-cell encapsulation from the brain tissue harvest, and dissociation was conducted in less than 1 h. 12–24-week-old mouse brains were rapidly dissected after CO_2_ anesthesia. The brain was sliced into 1 mm sections, and the hippocampus was extracted. The samples were enzymatically dissociated to obtain a single cell suspension with 0.05% papain and DNase1 in neurobasal medium at 37 °C for 30 min. After the enzymatic reaction was stopped by the addition of ovomucoid inhibitor albumin (Worthington Biochemical, LK003182), the cell suspension was passed through a 40 μm filter and washed by differential centrifugation to remove debris. The dissociated cells were added to Hank’s balanced salt solution containing 0.04% bovine serum albumin and diluted to 100 cells/μl.

### Cell loading and inDrop™ library preparation for scRNAseq analysis

Immediately after cell counting, a specific volume of cell suspension containing 5000 target cells was selected for further processing. A homemade microfluidic device was used to generate gel beads in the emulsion. The preparation of libraries was carried out using inDrop™ chemistry according to inDrop™ Library Preparation, Version 2.1 provided by the manufacturer. The quality and quantity of the library were assessed using a High-Sensitivity DNA Kit (Agilent Technologies, USA) on a 2100 Bioanalyzer (Agilent Technologies, USA). The sequencing process was performed on a NovaSeq6000 (Illumina, USA) or a NextSeq500/550 (Illumina, USA) in paired-end sequencing through the sequencing service companies (Macrogen, Korea/LAS, Korea).

### Quality control for scRNAseq analysis

The sequencing results were first processed through an inDrop™ sequencing data processing pipeline (https://github.com/indrops/indrops) based on Python 2.7 and aligned to a mouse genome reference (GRCm38 release 102). The datasets were imported into R environments via indRop (https://github.com/caleblareau/indRop) and used for subsequent bioinformatic analysis with the Seurat package (v4.2).

The initial dataset contained 147,371 barcoded beads with more than 1000 reads. Cells with fewer than 500 unique molecular identifiers filtered mapped (UMIFM) counts and genes with fewer than 10 cells were excluded, as low UMIFM indicates dead or dying cells. The R package scDblFinder was employed to identify and eliminate probable doublets. We further filtered cells whose nFeature_RNA and nCount_RNA were more than five standard deviations from the median value because a high nCount_RNA or nFeature_RNA implies that the cell may be a doublet or multiplet. In addition, cells with mitochondrial genes whose expression was more than two standard deviations above the median value of their total transcripts were eliminated from subsequent analysis. After the application of the above filters, the dataset contained a total of 22,233 cells.

### Data normalization and integration for scRNAseq analysis

Gene expression data for each cell were normalized and scaled using the Seurat *scTransform()* function, with regressing the number of genes and UMIs using *var.to.regress*. Using the Seurat workflow, the 1000 most highly variable genes were determined with the Seurat *SelectIntegrationFeatures* function. Genes were projected into a space defined by the principal components using the Seurat *RunPCA* function with the parameters of the variable genes. To integrate all the libraries, L2 normalization using the Seurat *RunHarmony* function was employed to correct for any possible batch effects. The top 25 variables obtained from the Harmony analysis were selected as inputs for dimension reduction by UMAP, shared nearest neighbor graph construction, and unbiased clustering using the Louvain method with multiple refinements.

### Cell annotation, and neuron sub-clustering for scRNAseq analysis

To identify the cell types comprising mouse hippocampal tissues, differential gene tests were performed on the datasets using the Wilcox Rank Sum test of the Seurat *FindAllMarkers* function. The top 10 marker genes with less than an adjusted p-value of 0.1 were used to manually assign cell type annotations for each cell cluster. In addition, we compared the cell types according to previously published marker genes. Out of 10 clusters, 2 clusters showed high expression of mouse neuron genes (*Meg3*, *Atp1b1*). 5468 mouse neurons were selected from the larger dataset based on cell type definitions, and their genes were projected into independent component space using the Seurat *RunICA* function. The 144 genes well-known as hippocampal canonical markers were utilized as the feature set for independent component analysis. To produce the new UMAP in neuron subsets, we performed the Seurat *RunUMAP* function with the following parameters: dims = 1:10, umap.method = umap-learn. There was no batch-dependent library effect. And then, the Seurat *FindNeighbors* and '*FindClusters*' functions were executed, as described above. This led to the identification of 9 neuron subclusters. We classified each subcluster of neurons based on their expression of canonical neuronal markers.

### Identification of differential expression genes and functional enrichment analysis

Differential expression genes in each cluster between WT and 5xFAD data were identified using the model-based analysis of single-cell transcriptomics implemented by the Seurat *FindMarkers* functions. Genes with a fold change greater than 0.25 and *p*-values less than 0.01 were considered differential expression genes. The differential expression genes were listed in the supplementary tables. For functional enrichment analysis, we used Enrichr in R to analyze the differential expression genes based on the biological process in the GO 2021 database. In addition, Cytoscape was used to generate a network of these genes.

### Statistical analysis

Statistical significance was analyzed using the GraphPad Prism (GraphPad Software Inc., USA). All data were presented as the mean ± standard error of the mean (S.E.M).

## Results

### Three-dimensional visualization of hippocampal axons in the Aβ plaque-rich environment

**Fig. 1:**
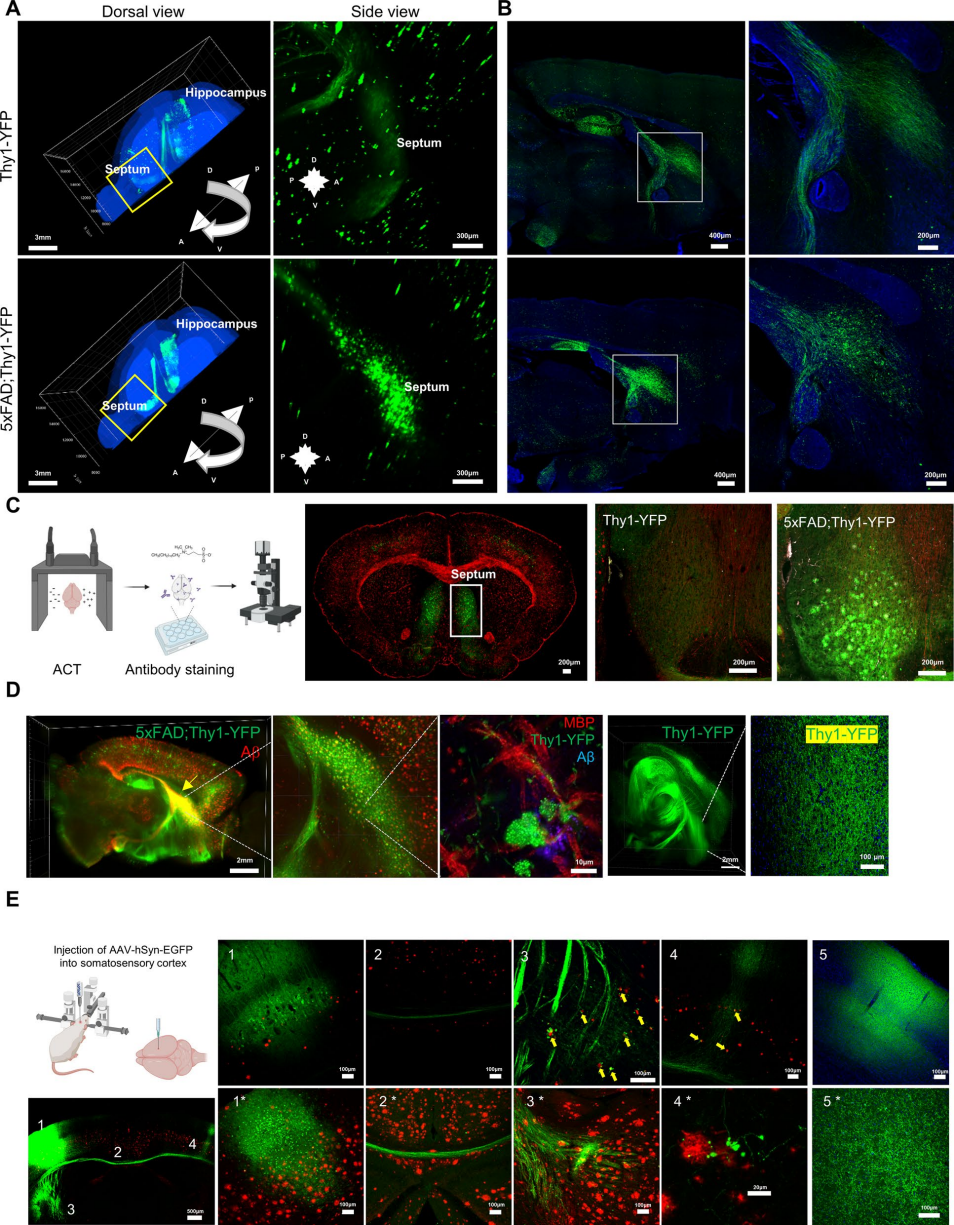
Axonal spheroids are formed in the hippocampo-septal tracts of 5xFAD; Thy1-YFP transgenic mice. **A** Whole-brain imaging revealed numerous axonal spheroids in the hippocampo-septal tracts of 5xFAD; Thy1-YFP transgenic mice. Scale bars = 3 mm for the dorsal view, 300 μm for the lateral view. **B** Sagittal sections and higher magnification images of the septum revealed that the transgenic mice had insufficient axonal fasciculation and a distinct morphology with a series of bulbous YFP-positive projections with enlarged ends. Scale bars = 400 μm for the left, and 200 μm for the right. **C** Schematic drawing for the active clarity method in conjunction with immunostaining and representative coronal sections showing aggregation of YFP-positive axons in the septum of 5xFAD; Thy1-YFP mice. MBP (red) was used to detect the axons in the sections. Scale bars = 200 μm. All schematic drawings were created with Biorender.com. **D** The presence and location of axonal spheroids in relation to amyloid plaques in the transgenic mice were studied using the ACT combined with immunostaining for Aβ (Yellow arrow: the septum, scale bar: 3 mm). Higher magnification images show that the axonal spheroids contained vesicular features (bright YFP signals in the spheroid) and were found at the end of MBP-stained axons around Aβ plaques. Whole brain imaging of Thy1-YFP WT mouse brain and higher magnification Thy1-YFP expression in the septum did not reveal any spheroids. Scale bars = 2 mm (whole brain), 10 μm (middle), 100 μm (rightmost). **E** A schematic drawing depicts the injection of AAV into the somatosensory cortex. A low magnification image under the drawing shows a tiled coronal image of AAV expression. The numbered magnified images are presented on the right panels with dendrite (1), callosal axons crossing the corpus callosum (2), corticothalamic axonal ends (3), and callosal axons reaching the contralateral cortex (4). AAV-hSyn-EGFP virus was injected into the somatosensory cortex of 6-month-old (1–4) and 8-month-old (1*–4*) 5xFAD mouse brains to determine the selectivity of neuronal compartments for spheroid development in the presence of Aβ plaques. The spheroid-like or bead-like big bulbous formations were not found in the somatic or dendritic compartments (1, 1*) or in a callosal bundle of nerve fibers in the corpus callosum (2, 2*). However, the spheroids near Aβ plaques were typically observed at the axon terminals of the ipsilateral corticothalamic route (3, 3*) and the callosal axons reaching the contralateral cortex (4, 4*; asterisk (*). AAV-hSyn-EGFP virus was injected into the somatosensory cortex of 8-month-old C57BL/6 mice (5) and the typical axon terminals of the ipsilateral corticothalamic route (5*) was presented (n = 4). Scale bars = 100 μm except for the leftmost image, scale bar = 500 μm and 4* = 20 μm

To determine the brain-wide distribution of DNs when Aβ plaques cause selective loss of synapses, we first acquired three-dimensional whole-brain imaging of 5xFAD (a widely used AD mouse model with mutations in *APP* and *PSEN1* genes leading to Aβ accumulation) mice with Thy1-YFP reporter at 6 months of age (5xFAD; Thy1-YFP). Specifically, the three-dimensional rendering of YFP images revealed neurite swelling in the form of spheroids, which were observed in the septum and the hippocampal regions of the brain (Fig. 1A, Additional file 1: Fig. 1A, B). Notably, the septum of transgenic mice displayed a dense and clustered appearance in contrast to the diffuse axon morphology in the Thy1-YFP wild-type (WT) mice (Fig. 1A, Additional file 2: Video 1A). In a sagittal section of the brain, differences in hippocampal axon morphologies were evident between the 5xFAD transgenic mice and Thy1-YFP WT mice. Enlargements within axon terminals with irregular spherical structures were observed more frequently in the septum of the 5xFAD mice than in the WT mice of the same age (Fig. 1B, Additional file 3: Video 1B). Additionally, axonal fasciculation was also deficient in the septum of 5xFAD mice, with each axon consisting of a series of bulbous regions in an aggregated form (Fig. 1B). In contrast, axons in the septum of WT control mice remained long, slender, and bundled. In addition to the findings in 6-month-old mice, we observed that Aβ plaques appeared simultaneously in the septal and cortical regions of 5-month-old mice. Similarly, neurite spheroids were detected in the septum and cortical regions of 5-month-old 5xFAD mice, while 5-month-old WT controls showed no evidence of altered or deformed morphology (Additional file 1: Fig. 1B).

### The Aβ accumulation was accompanied by the formation of spheroid axon terminals

To corroborate our histological findings that Thy1-YFP-expressing projection neurons in 5xFAD mice have neurite spheroids in the septal region, we utilized an immunostaining-compatible active clarity technique to specifically focus on the septum, where various types of hippocampal neurons project their axons [3, 24]. This technique allowed us to observe that axon terminals in the septal region of 6-month-old 5xFAD; Thy1-YFP transgenic mice exhibited numerous larger aggregates compared to those in WT control mice, as demonstrated by Thy1-YFP and myelin basic protein (MBP)-positive axons (Fig. 1C, D). Additionally, our results from the brain-wide imaging of 5xFAD mouse brains immunostained for Aβ showed that Aβ was distributed throughout the brain but was predominantly concentrated in the septum as well as the mammillary body with Thy1-YFP signals (Fig. 1D). High-magnification imaging of the Aβ and Thy1-YFP revealed that the neurite spheroids were adjacent to the Aβ fibrils but did not overlap. A high-resolution three-dimensional imaging of a neurite plaque showed that Aβ fibrils were characterized by a dense mass with a radiating halo, DNs with axon shafts adjacent to fibrils, and swollen spheroids with a vesicular structure (Additional file 4: Video 2). Overall, our results provide additional evidence that the majority of hippocampal axonal spheroids form adjacent to Aβ plaques in the septal region of 5xFAD mice.

### The formation of Aβ plaque-associated DNs at presynaptic neuron axon terminals

**Fig. 2:**
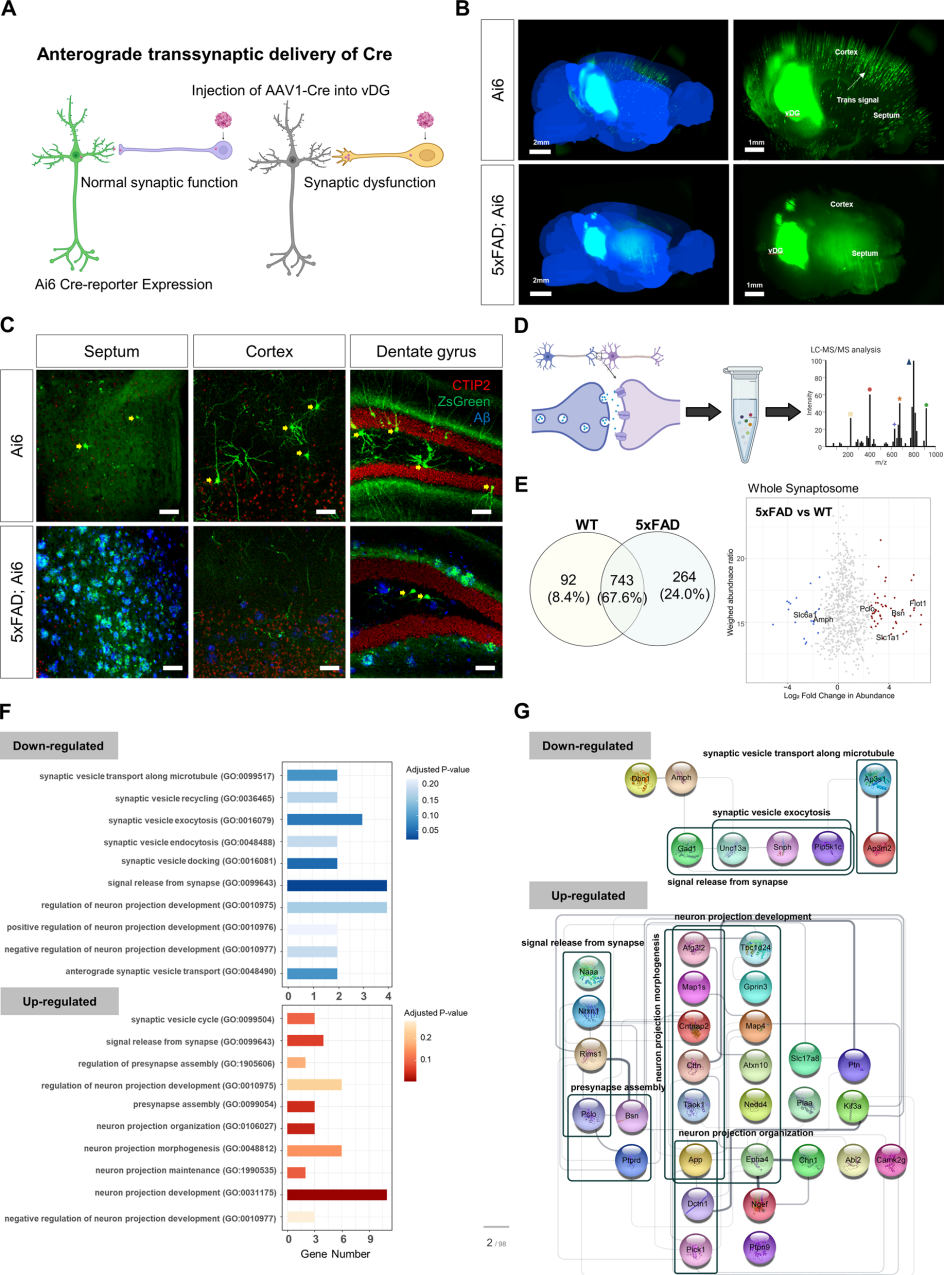
The formation of axonal spheroids in close proximity to axon terminals contributes to synaptic dysfunction due to anomalies in synaptic proteins involved in synaptic vesicle exocytosis. **A** To study the relationship between synaptic loss and axonal spheroid development, AAV1-hSyn-Cre was injected into the vDG of 6-month-old Ai6 control and 5xFAD; Ai6 transgenic mice. A schematic drawing depicts the Cre-Ai6 system used to record anterograde transsynaptic delivery events. **B** Whole-brain imaging of Ai6 and 5xFAD; Ai6 mice exhibited damaged synapses and reduced viral spreading across vDG connections in 5xFAD; Ai6 mice (bottom). Ai6 mice showed brain-wide spreading of Cre-mediated Ai6 expression (top). White arrow indicates Ai6 expression in the cortex, marked by transsynaptic transfer of AAV1-Cre. Scale bars = 2 mm (left), 1 mm (right). **C** Confocal images of coronal sections including the septum, hippocampus, and dentate gyrus of Ai6 and 5xFAD; Ai6 mouse brain immunostained for Aβ and Ctip2. In the septum, Ai6 mice showed transsynaptic labeling of a few neurons (yellow arrows) in contrast to the aggregation of Ai6 signals in the neuritic spheroids adjacent to Aβ plaques. In the cortex, Ai6 mice showed transsynaptic labeling of cortical neurons (yellow arrows), however, 5xFAD; Ai6 mice showed Ai6-positive neurites but did not show Ai6 labeled cortical neurons. In the dentate gyrus, Ai6-positive dentate granule neurons and hilus neurons were widely distributed, and a few Ai6-positive hilus neurons were visible in 5xFAD; Ai6 mice. Aggregation of Ai6 in the molecular layers depicts dystrophic axons near Aβ plaques. Scale bars = 100 μm. **D** Synaptosomes in the hippocampo-septal tracts of WT and 5xFAD mice were analyzed by LC–MS/MS to investigate the biological processes underlying axonal spheroid formation and synaptic dysfunction. **E** A total of 1,099 proteins, with 743 overlapping, 92 WT control-unique, and 264 5xFAD-unique proteins, were identified in the proteomic analysis of synaptosomes (Venn diagram). A scatter plot shows 337 up-regulated (log_2_ fold-change > 0.25, adjusted *p*-value < 0.1) and 109 down-regulated proteins (log2 fold-change < -0.25, adjusted *p*-value < 0.1) as a result of the differentially expressed protein analysis. Shown are the fold changes in protein abundance between 5xFAD (12-month-old, n = 4, 4 male) and WT (12-month-old, n = 4, 4 male) control synaptosomes and the weight value of this quantification. The position of the representative proteins selected for gene-set enrichment analysis is indicated in colors (blue = downregulated, red = upregulated). **F** Gene-set enrichment analysis based on GO Biological Process showed that both the downregulated and the upregulated proteins were linked to vesicle-related pathways, synaptic signaling pathways including chemical synaptic transmission and signal release from the synapse, and the neuron projection morphogenesis pathway. **G** PPI network analysis using the STRING database identified a network with 30 nodes and 50 edges from proteins in 10 upregulated pathways and a network with 20 nodes and 32 edges from proteins in 10 downregulated pathways, as shown in Fig. 2F

**Fig. 3:**
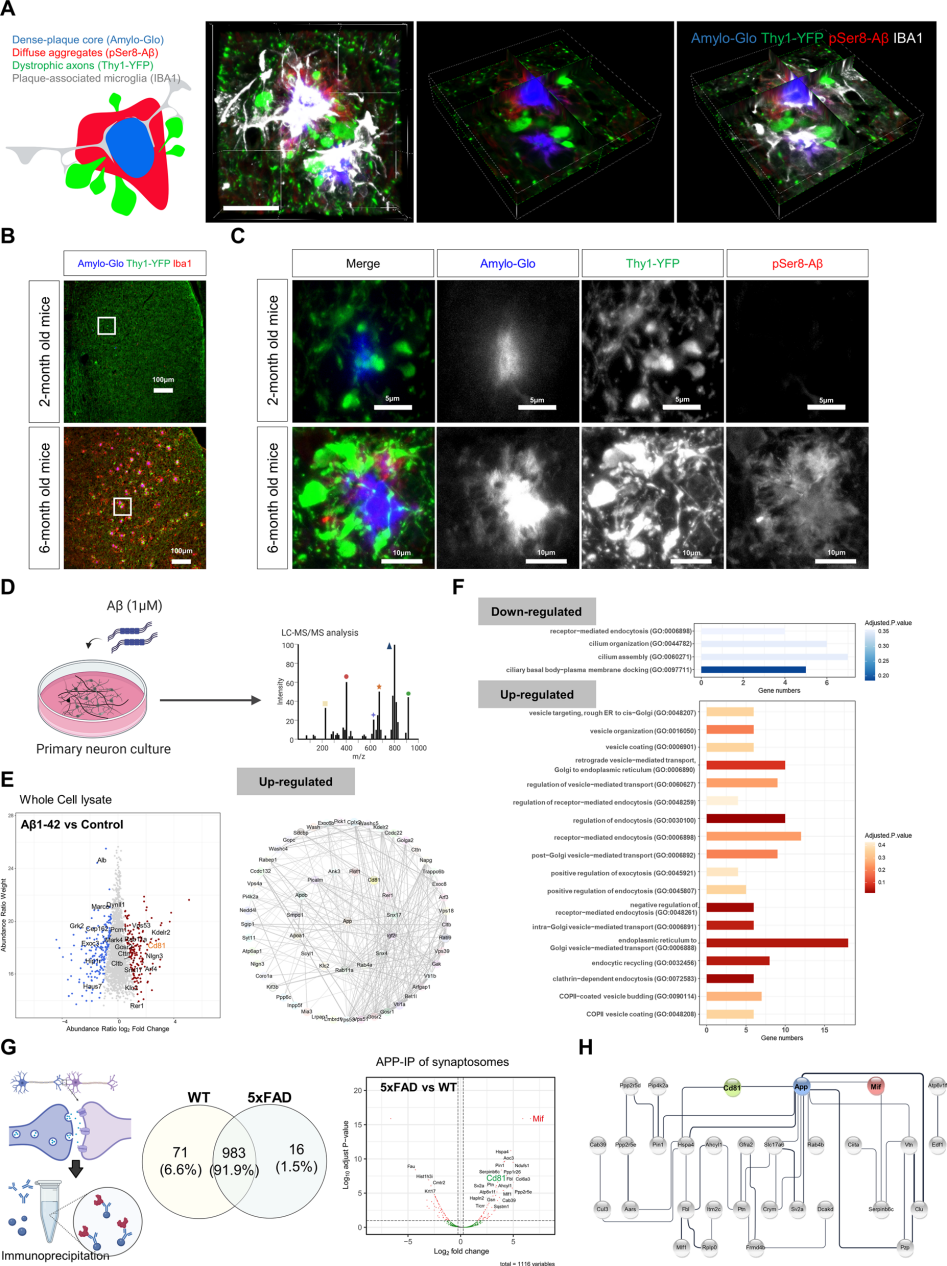
The enrichment and direct interaction of CD81, a tetraspanin protein of EVs, with APP in the synaptosomes of the 5xFAD mouse brain. **A** A schematic drawing depicts the localization of axonal spheroids and pSer8-Aβ (Aβ with phosphorylation at the Ser8 residue) in the periphery of an Amylo-Glo-stained plaque core. Plaque-associated Iba1-positive microglia create barriers in their processes. A representative three-dimensional image of axonal spheroids with Aβ plaques consisting of the core (Amylo-Glo), the diffuse area (pSer8-Aβ), and a microglial barrier (Iba1). Scale bar = 5 μm. **B** Confocal mages of the septum from 5xFAD; Thy1-YFP mice at two different ages (2 and 6 months) were immunostained for Iba1 and Aβ. Higher magnification images of the white box are presented in (c). Aggregation of YFP-positive axons and clustering of microglia are visible at 6-months of age. Scale bars = 100 μm. **C** Higher magnification images of the white box in (b) show that the axonal spheroids became larger as the amount of pSer8-Aβ increased. Scale bar = 5 μm for 2-month-old mice, 10 μm for 6-month-old mice. **D** Primary hippocampal neurons were harvested at 48 h after treatment with 1 μM Aβ1-42 synthetic oligomer for 6 h. Proteins isolated from the whole lysate of the three independent experiments (n = 3) were analyzed by LC–MS/MS. **E** A scatter plot shows 907 up-regulated (log_2_ fold-change > 0.25, adjusted *p*-value < 0.1) and 233 downregulated proteins (log_2_ fold-change < − 0.25, adjusted *p*-value < 0.1) as a result of the differentially expressed protein analysis. Shown are the fold changes in protein abundance between Aβ-treated and control primary hippocampal neurons and the weight value of this quantification. PPI network analysis using the STRING database identified a network of CD81, APP, and 65 other proteins with 310 interactions from the upregulated proteins of the differentially expressed protein analysis. **F** Gene-set enrichment analysis based on GO Biological Process showed 4 downregulated pathways related to cilia assembly and 18 upregulated pathways related to vesicle transport, endocytosis, and exocytosis. **G** The proteomic analysis of the APP C-terminal fragment-immunoprecipitated synaptosomes profiled the proteins involved in the initial formation of axonal spheroids during APP processing in the synaptosomes. A total of 1,070 proteins were identified with, 983 overlapping, 71 WT-unique, and 16 5xFAD-unique proteins. A volcano plot shows 519 up-regulated (log2 fold-change > 0.25, adjusted *p*-value < 0.1) and 307 down-regulated proteins (log2 fold-change < − 0.25, adjusted *p*-value < 0.1) as a result of the differentially expressed protein analysis. Shown are the fold changes in protein abundance between 5xFAD (6-months-old, n = 4, one female and three male) and WT (6-months-old, n = 4, two female and two male) synaptosome samples and the adjusted p-value. The positions of the representative proteins selected for further PPI analysis are annotated. **H** 29 PPIs were linked to the 38 proteins from synaptosomes that were immunoprecipitated with APP. Several proteins, such as MIF (red) and CD81 (green), interacted directly with APP (blue)

**Fig. 4:**
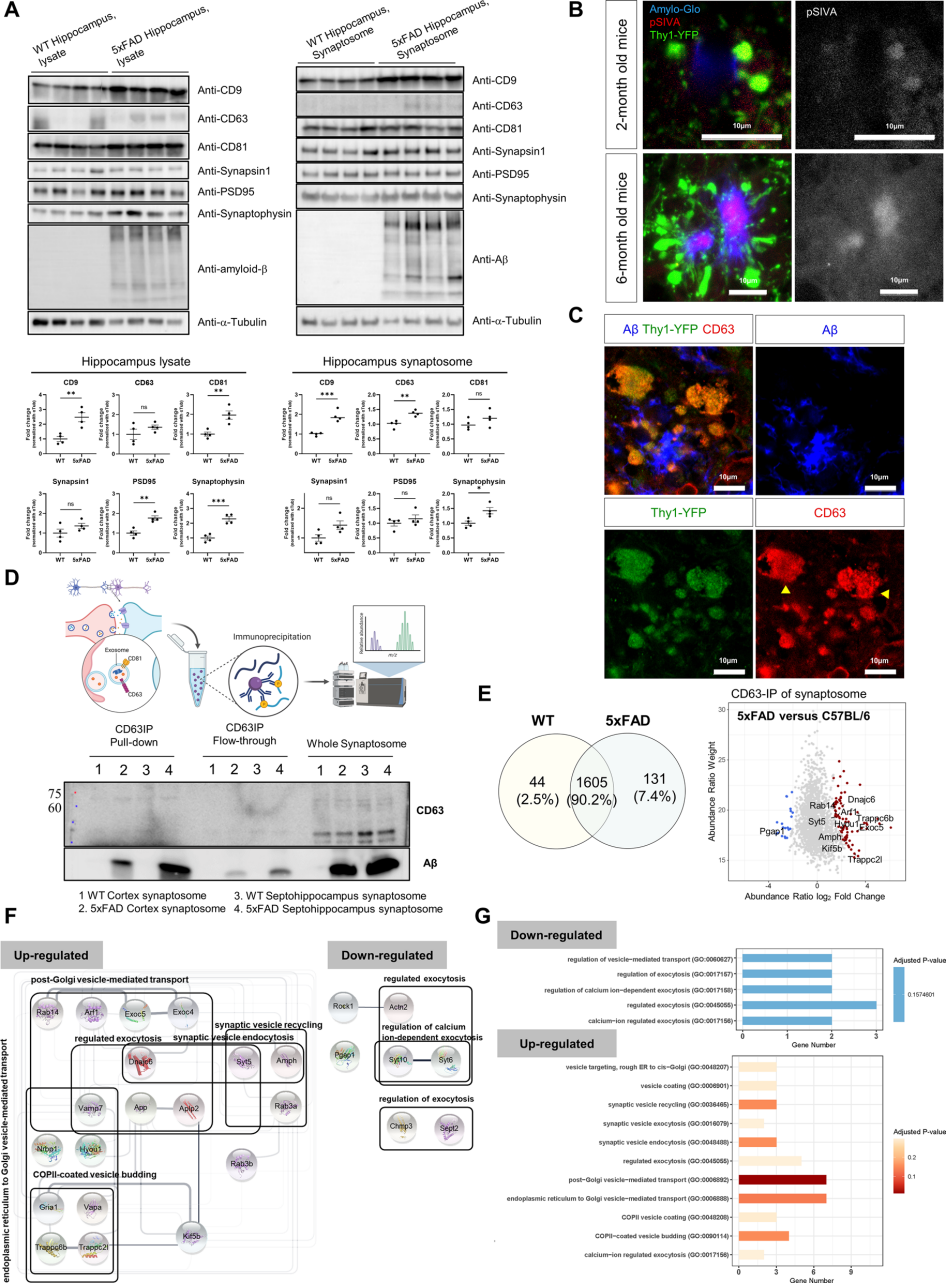
The accumulation of EVs in the axonal spheroids by dysfunctional vesicle exocytosis. **A** Tetraspanin proteins (CD9, CD63, and CD81), synaptic markers (Synapsin-1, PSD95: Postsynaptic density protein 95, and Synaptophysin), and Aβ protein were found in SDS-soluble lysates from hippocampal tissues and synaptosomes from 5xFAD (12-months-old, n = 4, four male) and WT control mice (12-months-old, n = 4, four male). 15 μg proteins from each sample were loaded. The plots are presented as the mean ± S.E.M. of four independent experiments. *p < 0.05; **p < 0.01; ***p < 0.001 as determined by the unpaired t-test. **B** Annexin-based biosensor called pSIVA, which detects exosome-like vesicles by binding to externalized phosphatidylserine was used to observe the accumulation of exosome-like vesicles at the axon terminals in the 5xFAD; Thy1-YFP mouse septum. pSIVA signals were detected in the DNs at 2-month-old mice but more localized in the Amylo-Glo-positive plaque core at 6-month-old mice. Scale bars = 10 μm. **C** Axonal spheroids surrounding the Aβ plaque of the 5xFAD; Thy1-YFP mice were fluorescently labeled for Aβ and CD63 to offer direct evidence of the accumulation of exosome-like vesicles at the axon terminals. Scale bars = 10 μm. **D** LC–MS/MS was utilized to identify CD63-immunoprecipitated proteins that were enriched in the synaptosomes collected from the hippocampo-septal tracts (hippocampo-septal synaptosome) of WT (6-months-old, n = 11, one female and ten male mice) and 5xFAD mice (6-months-old, n = 10, four female and six male mice) in order to explore the proteomic changes of EV states in the presence of Aβ plaques. The quality of the CD63-immunoprecipitated synaptosomal fractions was checked by western blot analysis of the pull-down, flow-through, and whole synaptosomes for CD63 and Aβ. **E** A total of 1,780 proteins were identified, including 1605 overlapping, 44 WT-unique, and 131 5xFAD-unique proteins. A scatter plot showed 211 up-regulated (log2 fold-change > 0.25, adjusted *p*-value < 0.1) and 65 down-regulated proteins (log2 fold-change < -0.25, adjusted *p*-value < 0.1) as a result of the differentially expressed protein analysis. Shown are the fold changes in protein abundance between 5xFAD and WT control CD63-IPed synaptosome samples and the weight value of this quantification. The positions of the significantly increased and decreased proteins are indicated. **F** The PPI network analysis using the STRING database identified a network with 19 nodes and 53 edges from the proteins in 11 upregulated pathways and a network with 7 nodes and 2 edges from the proteins in 5 downregulated pathways selected for gene-set enrichment analysis. **G** Gene-set enrichment analysis based on GO Biological Process showed 5 downregulated pathways related to vesicle-mediated transport and exocytosis and 11 upregulated pathways related to vesicle transport, endocytosis, and exocytosis

**Fig. 5:**
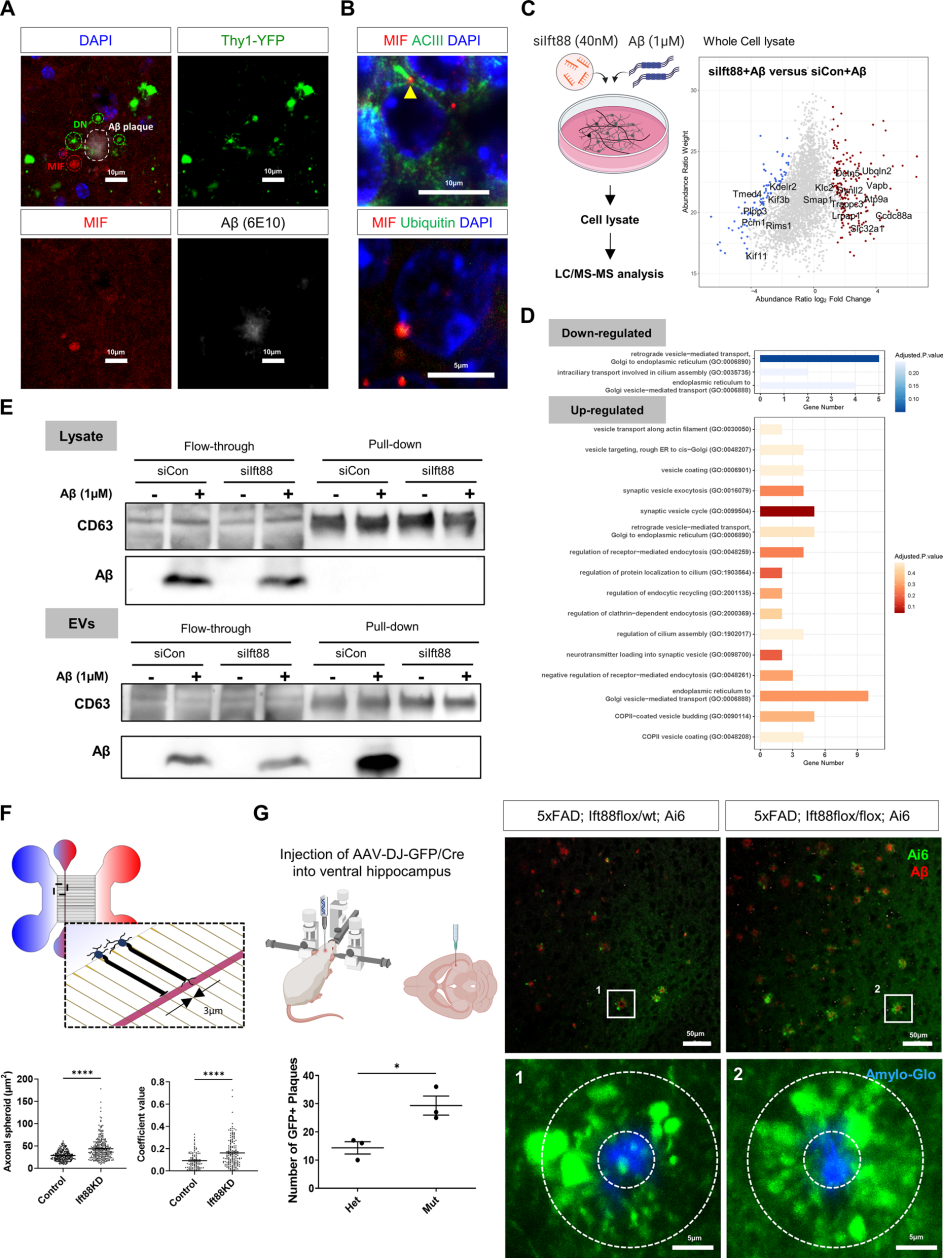
Primary cilia dysfunction contributed to axonal spheroid formation and Aβ deposition through vesicle accumulation. **A** Immunostaining of the 5xFAD mouse septum for nucleus (DAPI), Aβ, and MIF showed that MIF protein was located on DNs adjacent to Aβ plaques, suggesting that its failure of secretion and accumulation in DNs. Scale bars = 10 μm, green dashed lines: DN, white dashed lines: Aβ plaque, red dashed lines: MIF protein in DNs. **B** To examine the localization of MIF protein in the hippocampal neurons, MIF was immunostained using 5xFAD hippocampus. MIF protein was found at the basal end of ACIII-labeled primary cilia (yellow arrowhead) and co-localized with the ubiquitin-labeled proteasome system. Scale bar = 10 μm for ACIII and scale bar = 5 μm for ubiquitin staining. **C** Primary hippocampal neurons were harvested after siIft88 (40 nM) transfection for 48 h and oligomeric Aβ1-42 (1 μM) treatment for 6 h (siCon: scrambled short interfering RNA, siIft88: short interfering RNA for *Ift88*). Whole cell lysates (n = 3) were analyzed by LC–MS/MS. A total of 3546 proteins, with 3152 overlapping, 32 siCon-unique, and 362 siIft88-unique proteins, were identified. A scatter plot showed 527 up-regulated (log_2_ fold-change > 0.25, adjusted *p*-value < 0.1) and 126 down-regulated proteins (log_2_ fold-change < − 0.25, adjusted *p*-value < 0.1) as a result of the differentially expressed protein analysis. Shown are the fold changes in protein abundance between siIft88-transfected and siCon- transfected primary neurons and the weight value of this quantification. The position of the representative proteins selected for the pathway enrichment analysis is indicated. **D** Gene-set enrichment analysis based on GO Biological Process revealed 3 downregulated pathways related to vesicle-mediated transport and cilia assembly, and 16 upregulated pathways related to vesicle transport, endocytosis, and exocytosis. **E** Protein complexes were isolated from the primary neuron lysate (top) or the EV proteins collected from the conditioned media (bottom) followed by IP with the anti-CD63. Western blot analysis showed that Aβ was not complexed with CD63 in the lysate but was preferentially bound to CD63 in the control EVs. **F** Using hippocampal neurons derived from 5xFAD; Ift88-flox/flox mice grown in compartmentalized neuron culture platforms with Cre/loxP-mediated *Ift88* deletion, the area of axon terminals was measured (left plot, axonal spheroid), and the co-localization coefficients of CD63 and Aβ in the axon terminals were measured (right plot). Primary neurons were derived from 5xFAD mice to induce intraneuronal Aβ accumulation without extracellular treatment. Both axon terminal areas and co-localization of CD63 and intraneuronal Aβ were significantly increased in primary neurons with Cre-induced knock-down of *Ift88* expression (n = 3). Data are presented as the mean ± S.E.M. ****p < 0.001 as determined by the unpaired t-test (Ift88KD: knockdown of the *Ift88* gene). **G** 5xFAD; Ift88-flox/flox; Ai6 (mut) and 5xFAD; Ift88flox/wt; Ai6 (het) mice were used for stereotaxic injection of AAV-Cre. AAV vectors were injected into the ventral hippocampus, and confocal imaging was conducted using the septum to measure the number of axonal spheroids surrounding the Aβ deposition (n = 3). Higher magnification images were taken from the box area of the upper panel. Data are presented as the mean ± S.E.M. *p < 0.05 as determined by the unpaired t-test. Scale bars = 50 μm (upper), 5 μm (lower)

Our data so far have shown that DNs in the form of a spheroid were found at axon terminals proximal to Aβ plaques in the hippocampo-septal pathway of 5xFAD mice. Subsequent to the above observations, we next asked whether axons or dendrites are more prone to forming DNs. To examine the neuronal compartmental differences in forming neurite spheroids, we injected a GFP-expressing AAV under the control of a hSynapsin-promoter (AAV1-hSyn-GFP) into the somatosensory cortex of 6-month-old 5xFAD mouse brains (Fig. 1E). We examined two types of neurons that make callosal axons targeting the contralateral cortex and neurons that send axon output to the thalamus via corticothalamic axons. Interestingly, cortical neuronal dendrites projecting towards the meninges did not show any spheroids, and axon shafts in the corpus callosum did not show any dystrophic morphology of the spheroid-like or bead-like large bulbous structures. In addition, although Aβ plaques were distributed in the corpus callosum, callosal axons crossing the plaque were not affected (Fig. 1E2, 2*). Spheroids were found only at the axon terminals of the corticothalamic pathway and the callosal axons reaching the contralateral somatosensory cortex, indicating that neurite swelling was specific to axon terminals. These tendencies were stronger in the same location when AAV1-hSyn-GFP expression was prolonged in 8-month-old 5xFAD mice (Fig. 1E-1*– 4*). AAV1-hSyn-GFP expression in 8-month-old C57BL/6 did not show any aggregation of GFP signals (Fig. 1E-5, 5*).

### Aβ plaque-associated axonal spheroids impaired synaptic connection in the hippocampo-septal pathways

The loss of synapses, a key early event in AD pathology, has been traditionally assessed by counting abnormal dendritic spine morphology at postsynaptic terminals. However, our findings imply that synapse loss may be dramatically affected by axonal dystrophy at presynaptic terminals. To examine the association of synapse loss with axonal spheroid formation, an AAV1-mediated anterograde tracing system was utilized in Ai6 reporter mice expressing ZsGreen, a Cre reporter (Fig. 2A). AAV1-Cre was injected unilaterally into the ventral dentate gyrus (vDG) of both Ai6 mice and 5xFAD; Ai6 transgenic mice at 6 months of age. Cre-loxP system to record anterograde transsynaptic delivery of Cre recombinases showed the brain-wide distribution of Ai6 signals in WT Ai6 mice, confirming that hippocampal neuronal axons target various regions of the brain, including the cortex, contralateral hippocampus, and the septum (Fig. 2B, Additional file 5: Video 3A). Whole-brain imaging of the AAV1-Cre-injected 5xFAD mice revealed ZsGreen signals restricted to the injection site, with moderate ZsGreen expression in the septal area and low ZsGreen signal in the cortex, indicating that the infected virus was unable to cross the connections associated with the vDG neurons due to impaired synapses in the 5xFAD mice (Fig. 2B, Additional file 6: Video 3B). This result was confirmed in coronal brain sections of the septum, the cortex, and the dentate gyrus in Ai6 reporter mice. Coronal brain sections were immunostained for Aβ and Ctip2, which was used to counterstain both layer V cortical neurons and the dentate gyrus granule neurons. In the cortex and the contralateral dentate gyrus of WT Ai6 reporter mice, ZsGreen expressing neurons that received AAV1-Cre from the vDG neurons were evidently observed. A few septal neurons were also expressing ZsGreen (Fig. 2C). In 5xFAD; Ai6 mice, few neurons were labeled with ZsGreen in the cortex and the hippocampus of 5xFAD; Ai6 mice whereas the septum was heavily labeled with Ai6, suggesting that ZsGreen proteins accumulate in DNs proximal to Aβ plaques. This result demonstrated that axonal spheroids structurally impair the anterograde transmission of hippocampal projection neurons in 5xFAD mice, or that the impairment of axonal transport may influence DN formation (Fig. 2C).

The spread of toxic Aβ released in the peripheral area of compacted Aβ plaques could affect the formation of axonal spheroids. Staining of Aβ plaques in the septum and DG for presynaptic markers (STG1, Glut, Tau) and postsynaptic markers (Shank2, Map2) also revealed a connection between Aβ plaques and axons (Additional file 1: Fig. 2). STG1 and Tau proteins were co-localized with Aβ plaques and axon spheroids. Expression of Shank2, Map2, and dendrites as identified by spine-bearing YFP-positive neurites was excluded from the core of Aβ plaques. To identify the biological processes underlying these processes, mass spectrometry (MS)-based proteomics was performed on synaptosomes obtained from the hippocampo-septal pathway (Fig. 2D). We used special lysis buffer containing 8 M Urea, 2% SDS, 5 mM TEAB, and 150 mM NaCl to make proteins in the lipid domains soluble, which was not much considered in previous reports in using RIPA lysis buffer containing mild detergents (1% NP-40 and 0.1% SDS), one of general lysis buffer for protein analyses. By using the buffer, we discovered that proteins localized in the lipid domain, were enriched in the 5xFAD such as synaptosomes and hippocampal lysates, which were previously shown to be reduced in RIPA-soluble samples [58, 80]. Synaptosomal proteomics revealed that 1007 proteins and 835 proteins were detected in the brains of 5xFAD mice and WT controls, respectively. Differential protein expression analysis between synaptosomes of 5xFAD and WT control mouse brains demonstrated that 319 proteins were upregulated (log_2_ fold-change > 0.25, adjusted *p*-value < 0.1) and 112 proteins were downregulated (log_2_ fold-change < − 0.25, adjusted *p*-value < 0.1) (Fig. 2E, Additional file 7: Table 1). The differentially expressed proteins were studied further gene-set enrichment analysis utilizing the biological process of Gene Ontology (GO) (Fig. 2F) and protein–protein interaction (PPI) network analysis utilizing the STRING database (Fig. 2G). Gene set enrichment analysis based on the GO-BP database identified 480 and 690 pathways enriched in DNs without and with Aβ expression, respectively. To further explore the biological processes of DNs related to the synaptic function in the presence of Aβ, we filtered the pathways using specific keywords such as synaptic vesicle, signal release, neuronal projection, vesicle transport, and presynaptic assembly (Additional file 7: Table 1). Differentially expressed proteins showing associations with the dysfunction of synaptic vesicle-related pathways were highlighted. The proteins AP3M2 and AP3S1 were identified as deficient in 5xFAD and have been reported to play a role in synaptic vesicle transport along microtubules (GO: 0099517). In addition, UNC13A, PIP5K1C and SNPH were also found to be deficient in the 5xFAD mice and are known to play a role in synaptic vesicle exocytosis (GO: 0016079). The 5xFAD mice were found to be deficient in PIP5K1C and AMPH, which are known to be involved in the process of synaptic vesicle endocytosis (GO: 0048488). The 5xFAD-abundant proteins such as APP, AFG3L2, CNTNAP2, CTTN, MAP1S, and TAOK1 have been documented to participate in the process of neuron projection morphogenesis (GO: 0048812). These results suggest that Aβ accumulation may lead to the formation of axonal spheroids due to abnormalities in synaptic proteins involved in synaptic vesicle trafficking.

Interestingly, abnormalities in synaptic proteins involved in synaptic vesicle trafficking occurred prior to the accumulation of Aβ and the appearance of some axonal spheroids. This was demonstrated by our other experiments, which used MS-based proteomic profiling to analyze hippocampal lysates from 2-month-old C57BL/6 and 5xFAD mice. The results of the Western blot analysis showed that Aβ bands were present in the hippocampus of 2-month-old 5xFAD mice (Additional file 1: Fig. 3A) However, no prominent Aβ plaques bigger than 10 μm were observed in these 2-month-old mice, and the detection of DNs containing axonal spheroids was limited. Utilizing the identical mass spectrometry methodology employed in prior experiments, we successfully discerned a total of 958 distinct WT proteins, 12 exclusive 5xFAD proteins, and 925 proteins that were found to be common to both groups (Additional file 1: Fig. 3A). The differentially expressed proteins were 1,023 WT-abundant (log_2_ fold-change < − 0.25, adjusted *p*-value < 0.1, Additional file 8: Table 2) and 105 5xFAD-abundant proteins (log_2_ fold-change > 0.25, adjusted *p*-value < 0.1, Additional file 8: Table 2). The GSEA analysis based on GO-BPs was identical to the aforementioned analysis in Fig. 2, with 1040 pathways identified and 384 pathways retrieved using the same keywords (Additional file 1: Fig. 3B, Additional file 8: Table 2). The results of the MS-based proteomic profiling also showed that the expression pattern of proteins related to neuronal projection morphogenesis, which exhibited increased levels in the 5xFAD model as depicted in Fig. 2G, did not show significant expression in 5xFAD mice at the age of 2 months. However, the signaling pathways such as vesicle transport along microtubules, synaptic vesicle endocytosis, and synaptic vesicle exocytosis that were deficient in 5xFAD in Fig. 2G exhibited similar deficiencies in the mass spectrometry findings for 2-month-old mice (Additional file 1: Fig. 3C). Additionally, the corresponding AP3M2, AP3S1, PIP5K1C and SNPH proteins were also significantly depleted in the 5xFAD condition (Additional file 1: Fig. 3D). This suggests that the abnormalities in synaptic vesicle trafficking may be due to the Aβ expression, which may in turn contribute to the accumulation of Aβ and the formation of axonal spheroids in 5xFAD mice.

### Axonal spheroids surrounding Aβ plaques were affected by alterations in EVs in the axon terminals

Neuronal exocytosis may be associated with the formation of axonal spheroids, we next investigated the role of Aβ in axonal spheroids by imaging and proteomic analysis. Examination of three-dimensional images of axonal spheroids in the septum of 5xFAD; Thy1-YFP brain near Aβ plaques showed Iba1-positive microglial processes surrounded Amylo-Glo-labeled Aβ core, and Ser8-phosphorylated Aβ (pSer8 Aβ) was detected in Amylo-Glo periphery. YFP-positive axons were found adjacent to Aβ fibrils, whereas the majority of spheroids were found outside of Aβ fibrils. A small number of microglial processes were in contact with axon spheroids, which could be indicative of the phagocytosis of DNs by microglia. (Fig. 3A, Additional file 4: Video 2). Thy1-YFP-expressing DNs and pSer8 Aβ-stained diffuse aggregates were localized in the periphery of Aβ plaques, whereas plaque-associated microglia were more frequently co-localized with Amylo-Glo-stained dense-core plaques than the DNs. Also, sections from two different ages of 5xFAD; Thy1-YFP mice demonstrated that the morphology of axonal spheroids became more obviously inflated as the amount of Aβ and pSer8 Aβ increased (Fig. 3B, C). During the initial phases of Aβ plaque development in the septum of 2-month-old mice, YFP-positive axons displayed densely innervating patterns, with some spheroids appearing in close proximity to Amylo-Glo-stained Aβ plaques. By 6 months of age, spheroidal axons had accumulated in greater numbers surrounding the Amylo-Glo-stained plaques. Together, our findings indicate that Aβ fibrils grow adjacent to axons and promote the formation of axonal spheroids by pSer8 Aβ expansion.

We then examined the proteomic alteration caused by Aβ treatment in cultured hippocampal neurons. The primary hippocampal neurons were treated with freshly solubilized Aβ1-42 for 6 h at a final concentration of 1 μM on five days after plating. After a fresh medium including 1 μM of Aβ1-42 was used to replace the preceding medium, the neurons were incubated for an additional 48 h prior to being subjected to mass spectrometry combined with liquid chromatography (LC–MS/MS) analysis (Fig. 3D). Proteomic analysis of cell lysates revealed 744 distinct proteins in Aβ1-42-treated neurons, 48 distinct proteins in vehicle-treated neurons, and 2,440 proteins in both groups. Differential protein expression analysis between Aβ1-42-treated and vehicle-treated neurons found that 907 proteins were upregulated (log_2_ fold-change > 0.25, adjusted *p*-value < 0.1) and 233 proteins were downregulated (log_2_ fold-change < − 0.25, adjusted *p*-value < 0.1) (Fig. 3E, Additional file 9: Table 3). The PPI network analysis discovered that APP expression was associated with 66 other proteins in the Aβ1-42-treated neurons. Furthermore, we found that CD81 proteins were induced along with other vesicle-associated proteins such as SDCBP, RAB11A, SMPD1, and FLOT1 by Aβ treatment. Gene-set enrichment analysis based on the GO biological database demonstrated that Aβ1-42 treated neurons activated pathways involved in synaptic vesicle endocytosis or vesicle transport. Interestingly, the same analysis showed that cilia assembly-related pathways were downregulated in neurons treated with Aβ1-42 (Fig. 3F, Additional file 9: Table 3). These results suggest that Aβ may interfere with the normal function of cilia as well as endocytosis and vesicle transport within the neuron.

Accordingly, we hypothesized that neuronal proteins that interact with APP might have a significant impact on the formation of axonal spheroids. APP was immunoprecipitated from hippocampo-septal synaptosomes of 5xFAD and WT control mice, and the immunoprecipitated synaptic proteins were identified by LC–MS/MS (Fig. 3G). We identified 1,070 unique proteins from the APP-immunoprecipitated synaptic proteins, with 519 upregulated proteins (log_2_ fold-change > 0.25, adjusted *p*-value < 0.1) such as MIF, SLC17A6, and CD81, and 307 downregulated proteins (log_2_ fold-change < − 0.25, adjusted *p*-value < 0.1) such as ARF6, PRKCE, and PPP3R1 (Additional file 10: Table 4). Notably, PPI network analysis of upregulated proteins using the STRING database identified APP interaction targets such as PIN1, HSPA4, SLC17A6, RAB4B, CLU, VTN, and MIF in a network with 38 nodes and 39 edges (Fig. 3H). In addition, Syn-GO gene-set enrichment analysis of these results identified regulation of presynaptic dense core vesicle exocytosis and regulation of synaptic vesicle cycle as down-regulated proteins, while neuronal dense core vesicle was identified an up-regulated proteins (Additional file 1: Fig. 4). This prediction that the transgenic expression of APP would lead to an increase in synaptic dense core vesicles is consistent with the recent study that APP accumulated with presynaptic proteins around amyloid plaques [32]. Also, our Syn-GO gene-set enrichment analysis [38] identified that the pathways related to vesicle cycling or exocytosis were suppressed in 5xFAD mice. This finding was in line with our previous proteomic analysis, reporting abnormalities in vesicle exocytosis associated with the development of axonal spheroids near Aβ plaques.

### Accumulation of EVs and formation of axonal spheroids in 5xFAD hippocampal neurons

Previously, we showed that CD81 was identified as an interactome of Aβ and that some pathways involved with synaptic vesicles were altered in the synaptosome of 5xFAD mice, suggesting that different types of exocytic vesicles might be accumulated in the axonal spheroids of 5xFAD mice. To determine which vesicles were mainly enriched in axonal spheroids with Aβ over-expression, western blot analysis was used to assess the expression levels of common EV markers (CD9, CD63, and CD81) (Fig. 4A, Left). To solubilize proteins accumulated in DNs, we dissolved protein extracts in 5% SDS for the analysis. Our western blot analysis revealed a significant increase in CD81 and CD9 proteins in hippocampal whole cell lysates and a significant upregulation in CD9 and CD63 proteins in hippocampal synaptosomes of 5xFAD mice, which indicates accumulation of EVs in the 5xFAD brain.

In light of a recent study showing that EVs containing CD63 together with one or the other two tetraspanin proteins were likely to be formed from endosomes [48], these findings provide insight into the accumulation of endosome-derived EVs or multivesicular bodies in axonal spheroids near Aβ plaques. Interestingly, our western blot analysis of hippocampal synaptosomes showed that the CD81 protein, which we have shown to interact with Aβ, and the CD9 protein were mainly detected in the 5xFAD mice compared to the WT mice. The results imply that CD81-containing EVs, multivesicular bodies, and CD9-positive small EVs derived from the plasma membrane could accumulate in the DNs [39, 44]. The western blot analysis for synaptophysin, a marker for small synaptic vesicles, revealed that axonal spheroids in 5xFAD mice may accumulate synaptic vesicles, as indicated by the significant enrichment of synaptophysin in the synaptosome (Fig. 4A, Right). Our study also showed that the difference between 5xFAD and WT was observed in synaptophysin expression, which might reflect the increased proportion of synaptophysin in the urea/SDS-soluble membrane compartment in 5xFAD mice. And no significant difference in the levels of the postsynaptic marker PSD95 was observed between the two genotypes. These results support the production of axonal spheroids by the accumulation of presynaptic EV proteins.

Next, we examined the accumulation of EVs in DNs by immunostaining the septum of the 5xFAD; Thy1-YFP mouse brain. An annexin-based biosensor known as pSIVA was used to detect exosome-like vesicles in the extracellular environment. pSIVA is a polarity-sensitive indicator of viability and apoptosis that binds to externalized phosphatidylserine and is a sensor for exosome-like vesicles, which are frequently characterized by an abundance of cholesterol, glycosphingolipids, and phosphatidylserine [69]. A few axonal spheroids and a very faint pSIVA signal were detected in the septum of a 2-month-old 5xFAD; Thy1-YFP transgenic mouse, indicative of the initiation of EV accumulation in DNs (Fig. 4B). In the septum of the 6-month-old 5xFAD; Thy1-YFP mice, a strong pSIVA signal was detected clustered with DNs and Amylo-Glo. In contrast, no pSIVA signal was detected near axon terminals without axon spheroids. Direct evidence for the accumulation of exosome-like vesicles at the axon terminals was provided by fluorescent labeling of axonal spheroids in 5xFAD mice (Fig. 4C). CD63 protein was found in the same location as axonal spheroids close to Aβ plaques. These results support the idea that the vesicle-like structures in DNs (Fig. 1C, D) were accumulated from endosomes and multivesicular bodies.

Given that we found CD63-positive vesicles accumulated in axonal spheroids around Aβ plaques, we further investigated how they spread into the hippocampo-septal axon tract. To do this, we stereotactically injected an AAV vector expressing EGFP-tagged CD63 (AAV-CAG-CD63-EGFP) into the vDG to trace the location of CD63-EGFP-positive EVs in the brain of 5xFAD mice (Additional file 1: Fig. 5A). When the whole brains of WT mice were observed using a low-power lens, CD63-EGFP-expressing EVs were seen at the injection site and the contralateral vDG (Additional file 1: Fig. 5B). In contrast, CD63-EGFP expression was seen throughout the brain of 5xFAD mice, where CD63-EGFP-expressing EVs were aggregated at a size detectable with a low numerical aperture lens. This clustering of vesicles is consistent with our previous findings that Aβ caused axonal spheroid formation and synaptic disruption. Whole brain images acquired with the active clearing technique [43] confirmed our findings and showed that the distribution of CD63-EGFP-expressing EVs formed aggregations in the septum of 5xFAD mice (Additional file 11: Video 4_WT, Additional file12: 5xFAD). The septal region was further examined by immunohistochemistry. The expression of CD63-EGFP in WT mice was widely distributed in the extracellular area (Additional file 1: Fig. 5C). On the other hand, in the septum of 5xFAD mice, a dense core of Aβ plaques was grouped with DNs expressing CD63-EGFP. A closer inspection of axonal spheroids in the septum of 5xFAD mice revealed that CD63-EGFP was co-localized with the APP-expressing DNs (Additional file 1: Fig. 5D). These results suggest that the DNs contain accumulated EVs.

To evaluate how the status of these vesicles was changed with Aβ expression, we performed proteomic analysis for the proteins immunoprecipitated with CD63, which were enriched in the synaptosome of 5xFAD mice (Fig. 4D). Fractions of the synaptosomes immunoprecipitated with CD63 were western blotted for CD63 and Aβ to monitor the quality of the fractions. For CD63, a slight band was detected in all samples of the immunoprecipitation (IP) group and the whole synaptosome group, but not in the fractions of the flow-through group. Aβ was more prevalent in the synaptosomes obtained from the hippocampo-septal tissues of the 5xFAD mice than in the cortical synaptosomes. Proteomic analysis identified a total of 1780 proteins, of which 65 were upregulated and 211 were downregulated in synaptosomes immunoprecipitated with CD63 from 5xFAD hippocampo-septal tracts (Fig. 4E, Additional file 13: Table 5). In order to better describe the interactome of CD63, we constructed PPI networks using the STRING database. Of the 65 proteins abundant in 5xFAD synaptosomes, our network analysis identified 21 nodes with 52 edges, with APP being the most prominent (Fig. 4F). This finding that APP was the primary interactome of CD63 suggested that EVs might aggregate at axon terminals in APP-expressing neurons, resulting in axonal spheroids, as reported in the previous study [13]. In addition, we applied the 65 proteins upregulated and the 211 proteins downregulated in 5xFAD mice to the gene-set enrichment analysis based on GO signatures in the biological process (Fig. 4G, Additional file 13: Table 5). CD63 was found to interact with proteins that helped with vesicle-mediated transport, synaptic vesicle endocytosis, and vesicle budding. This shows that the synaptosomes of the 5xFAD mice possess proteins that normally enable vesicles to function. Several proteins involved in the regulated exocytosis pathway were downregulated in the synaptosomes of 5xFAD mice when CD63 was also co-expressed. In particular, the downregulated charged multivesicular body protein 3 (Chmp3) protein was linked to endosome transport via the multivesicular body sorting pathway (GO:0032509) or endosome-to-lysosome transport via the multivesicular body sorting pathway (GO:0032510) among GO signatures. Intraneuronal Aβ42 accumulates prominently in the multivesicular body (MVB) of neurites and presynaptic compartments, which precedes the appearance of Aβ in extracellular Aβ plaques [18, 72]. Taken together, our results suggest that intraneuronal transport of Aβ may occur via vesicles containing CD63 and accumulate at axon terminals, forming spheroid by extracellular Aβ plaques.

### Knockdown of *Ift88* expression in hippocampal neurons exacerbated axonal dystrophy

The increased expression of the macrophage migration inhibitory factor (MIF) protein (Fig. 4G, H), which is encoded by the *Mif* gene, provided additional evidence for the previous findings that an inflammatory response is closely associated with Aβ plaques [51, 85]. MIF is elevated in the AD brain and mediates pro-inflammatory signaling by promoting the secretion of IL-6 and TNF-α [46]. Even though neurons express MIF, the function of neuronal MIF has not been identified yet. There has been a previous study showing that MIF co-localized with Aβ plaques in APP23 transgenic mice, suggesting an association between MIF and AD [85]. This spotted expression pattern of MIF may suggest an accumulation of neuronal MIF in the dystrophic axon terminals. To confirm aggregation of MIF in the hippocampal neuronal axon terminals, we stained MIF in the septum of 5xFAD; Thy1-YFP mice. Indeed, the immunostaining of the septum in 5xFAD mice showed that MIF protein was detected on dystrophic axons proximal to Aβ plaques, supporting its role as a cytokine at axon terminals and possible interaction with the neuronal APP (Fig. 5A). In the hippocampus, MIF protein was localized to the basal end of type 3 adenylyl cyclase (ACIII)-labeled primary cilia, suggesting that it might serve as a chaperone protein in the proteasome complex. The accumulation of MIF in the perinucleus was co-stained for ubiquitin marking the localization of MIF chaperone proteins in the proteosome complex. These results suggest that neuronal clearance may play a role in axonal dystrophy and the accumulation of EVs in the axon spheroids via the primary cilia (Fig. 5B). These findings are in line with current evidence that MIF regulates ciliary gene transcription by binding to the promoters of the IFT-A (*Ift121*, *Ift139*, *Ift140*, *Ift144*) and IFT-B (*Ift27*, *Ift88*) genes and plays a critical role in cilia formation and elongation [84].

To evaluate the role of primary cilia in axonal dystrophy such as axonal spheroid formation, we downregulated the expression of the Intraflagellar transport protein 88 (*Ift88*) gene in the primary hippocampal neurons. The deletion of the *Ift88* gene causes primary cilia to be unable to form or function correctly via inhibition of an IFT-B complex [14, 59]. Transfection of primary hippocampal neurons with 40 nM short interfering RNA (siRNA) against *Ift88* (siIft88) for 48 h resulted in reduced Ift88 expression levels compared to neurons transfected with control siRNA (siCon). Western blot analysis also showed that CD63 levels remained stable, whereas CD81 levels decreased in neuronal extracts (Additional file 1: Fig. 6A) and increased in EVs obtained from the conditioned media (Additional file 1: Fig. 6B). Taken together, *Ift88* silencing greatly induced the release of CD81-containing EVs from hippocampal neurons. To study the function of *Ift88* in neurons exposed to Aβ, we analyzed neuronal lysates and EVs derived from primary neurons that were transfected with 40 nM siIft88 for 48 h and treated with 1 μM Aβ for 6 h. Our findings showed that knockdown of *Ift88* slightly induced Aβ in the cell lysates (Additional file 1: Fig. 6C). This indicates that dysfunction of primary cilia may further enhance Aβ uptake or inhibit neuronal clearance. To get more specific evidence for the uptake of Aβ, we investigated low-density lipoprotein receptor-related protein 1 (LRP1), which is an endocytic receptor and binds Aβ for neuronal uptake [34], in the septum of 5xFAD. LRP1 was found to be highly expressed in the swelling compartment of DNs (yellow dashed lines in Additional file 1: Fig. 6D), suggesting that primary cilia may be involved in the uptake of extracellular Aβ by neurons.

LC–MS/MS was employed to identify proteomic alterations in primary neurons that were transfected with siIft88 and treated with Aβ. Proteomic profiling of whole cell lysates identified 127 downregulated proteins (log_2_ fold-change < − 0.25, adjusted *p*-value < 0.1) and 527 upregulated proteins (log_2_ fold-change > 0.25, adjusted *p*-value < 0.1) in the Aβ-treated neurons after *Ift88* knockdown (Fig. 5C, Additional file 14: Table 6). Among these differentially expressed proteins, intra-ciliary transport proteins involved in ciliary assembly, such as KIF3B and PCM1, were significantly deficient in the Aβ-treated neurons after Ift88 knockdown. The endocytosis-related proteins such as LRPAP1, SMAP1, and UBQLN2, the vesicle-mediated transport proteins such as KLC2, ATP9A, VAPB, DCTN5, and DYNLL2, were abundant in the Aβ-treated neurons after Ift88 knockdown. GO biological process pathway analysis revealed that the differentially expressed proteins were assigned to 964 pathways, and among these pathways, vesicle trafficking, endocytosis, and exocytosis were significantly altered by decreased *Ift88* (Fig. 5D, Additional file 14: Table 6). These findings suggest that primary cilia dysfunction affects vesicles to transport Aβ via a vesicle-mediated trafficking mechanism or vesicle-secretion mechanism.

To investigate how Aβ is processed by EVs in neurons, CD63 IP was performed using lysates and EVs obtained from neurons that were transfected with siIft88 and treated with Aβ (Fig. 5E). Western blot detection of Aβ was performed on flow-through samples of cell lysates and EVs. The results showed that Aβ was present in neurons and their EVs after Aβ treatment, independent of transfection with siIft88, suggesting that Aβ was able to enter neurons and be transported by EVs. Western blot signals resulting from the use of CD63 were detected and those resulting from the use of Aβ were abolished in all pull-down samples of cell lysates, indicating that most of CD63-containing vesicles with Aβ in Aβ-treated neurons were secreted from these neurons. Interestingly, when we treated Ift88-silenced neurons with Aβ, Western blot signals for Aβ disappeared in CD63 IP samples of EVs from these neurons, while those for CD63 were maintained in all samples. The results showed that Aβ transported by EVs in the neurons that had undergone primary ciliary dysfunction was infrequently secreted from these neurons and could accumulate in the axon terminals of neurons, forming axonal spheroids.

To elucidate that the dysfunction of primary cilia promotes axonal spheroid formation through vesicle accumulation, we used a Cre/LoxP-mediated Ift88 deletion technique to silence *Ift88* expression in hippocampal neurons cultured in microfluidic chambers (Fig. 5F, Additional file 1: Fig. 7). The hippocampal neurons were cultivated using embryos obtained from breeding 5xFAD; Ift88-flox/flox conditional mice. The neurons were infected with an AAV vector expressing GFP and Cre recombinase (AAV-DJ-GFP/Cre) or a similar viral construct containing GFP for 48 h at 7 days after plating neurons and kept for an additional 9 days in the microfluidic chamber. Notably, confocal images with Aβ staining confirmed that the area size of axon terminals with Aβ deposition was dramatically increased in the neurons infected with AAV-DJ-GFP/Cre but not those infected with AAV-DJ-GFP. In addition to Aβ staining, and in line with the dysfunction of vesicle exocytosis, we observed co-localization of CD63 staining with Aβ deposition at axon terminals. The colocalization coefficients of CD63 and Aβ were significantly improved in the primary neurons with the conditional knockdown of *Ift88* expression. To confirm the primary cell culture findings in a more biologically relevant milieu, we next injected AAV-Cre into the ventral hippocampus of conditional Ift88-flox/flox *or* Ift88-flox/wt mutant mice carrying 5xFAD and Ai6 transgenes. ZsGreen was detected in the ventral hippocampus and the septum of the AAV-infected mice (Fig. 5G). The number of Ai6-positive plaques with Aβ staining (White box in Fig. 5G) was significantly higher in the Ift88-flox/flox homozygotes than the heterozygotes after Ift88 knockdown. These data provide direct evidence that primary cilia dysfunction contributes to the secretion of CD63-containing vesicles containing Aβ. This leads to axonal spheroid formation through vesicle accumulation in DNs of exocytosis-compromised neurons.

### Single cell transcriptome profiling on the hippocampus uncovered that specific types of neurons were vulnerable to Aβ expression and EV secretion

Since Aβ was delivered in CD63-containing vesicles and the vesicles accumulated at the axon terminals by Ift88 dysfunction, as shown in our results, we thought that axonal spheroid formation might occur in a specific type of neuron with long-distance projections and impairments in EV secretion. We therefore used the inDrops™ platform to sequence 8 libraries: 5 libraries from the hippocampus of male AD mice at 12 and 24 weeks of age, and 3 libraries from the hippocampus of age-matched WT mice for the purpose of finding the specific vulnerable neuron types. 22,233 cells with 36,090 transcriptomes were collected after quality control was completed. We integrated our data with a public dataset (GSE129788) as a reference mapping using L2 normalization to determine the precise cell type classification. Individual cells were viewed in uniform manifold approximation and projection (UMAP) dimensions (Additional file 1: Fig. 8A). We annotated 10 main cell types by identifying cluster-specific markers from the comparison of their expression in the given cluster to that in the others (Additional file 1: Fig. 8B) and by matching known marker genes from the literature in the reference dataset.

**Fig. 6:**
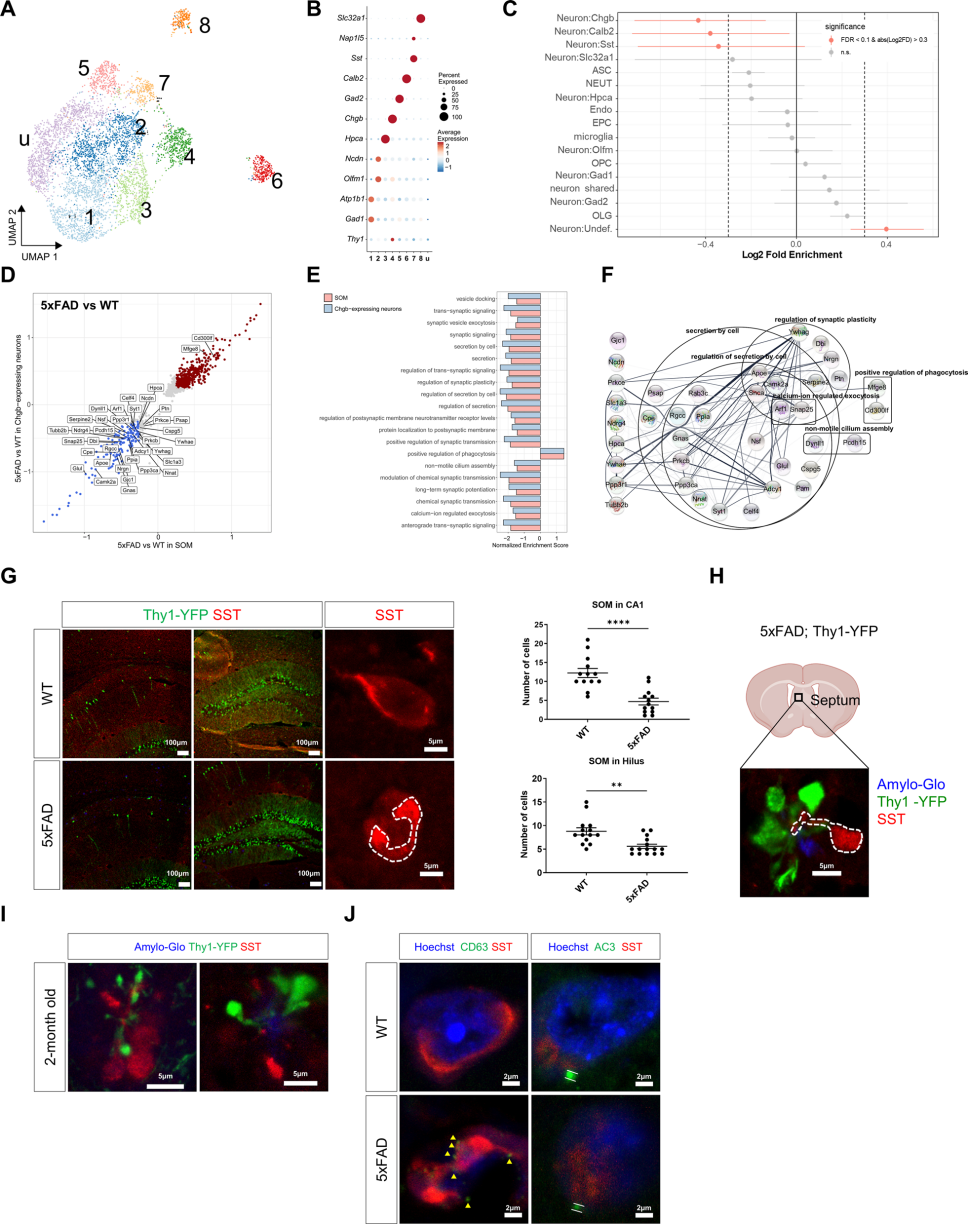
Single-cell transcriptome profiling of the hippocampal tissues identified the specific neuron type associated with vesicle accumulation in the septum. **A** A UMAP plot shows the subtypes of neurons produced from WT (3-month-old, n = 3, 3 male; 6-month-old, n = 1, female) and 5xFAD (3-month-old, n = 4, male; 6-month-old, n = 2, female) hippocampus tissues. Using independent component analysis using canonical markers, nine different neuronal subclusters, including 1 undefined (u) were found from 5468 neurons, characterized by strong expression of *Meg3* and *Atp1b1*. Each cluster is color-coded according to cell type. **B** A dot plot illustrated the expression of selected marker genes in each neuron subtype (u: undefined). **C** A permutation plot to calculate the average proportion of each neuron subtype between WT and 5xFAD mouse hippocampal tissues demonstrated that the number of neurons with *Sst*, *Chgb*, and *Calb2* markers was significantly decreased in 5xFAD mice (ASC: astrocyte, Endo: endothelial cells, EPC: ependymal cells, NEUT: neutrophils, OPC: oligodendrocyte precursor cells, OLG: oligodendrocytes). **D** Differential gene expression analysis revealed 620 up-regulated (log_2_ fold-change > 0.25, *p*-value < 0.01) and 234 down-regulated genes (log_2_ fold-change < − 0.25, *p*-value < 0.01) in the SOM, and 738 up-regulated (log_2_ fold-change > 0.25, *p*-value < 0.01) and 186 down-regulated genes (log_2_ fold-change < -0.25, *p*-value < 0.01) in the *Chgb*-expressing neuron. Shown are the fold changes of gene expression between 5xFAD and WT control mouse hippocampus in SOM and the fold changes of gene expression between 5xFAD and WT control mouse hippocampus in *Chgb*-expressing neurons. **E** Gene-set enrichment analysis in GO biological process signatures showed that SOM and *Chgb*-expressing neurons commonly turned down pathways related to secretion, cell secretion, and the regulation of cell secretion, as well as the non-motile ciliary assembly pathway. **F** Gene network connections and associated functions were visualized based on GO biological process signatures derived from differential gene expression analysis of SOM. Gene lists for gene ontology terms are derived from SOM, as shown in Fig. 6E. The genes related to secretion, non-motile cilium assembly, phagocytosis, and synaptic plasticity were enclosed by black boxes. The gene symbols were matched with the STRING database, and we identified a network with 39 nodes and 132 edges. **G** Immunofluorescence analysis of hippocampal sections from WT and 5xFAD; Thy1-YFP mice showed that the number of SOM in the CA1 area of the hippocampus (left), and the dentate gyrus hilus (middle) was lower in 5xFAD mice than in WT. Plots represent the number of SOMs in each area. Higher magnification images of SST expression showed a bright aggregation of SST in the perinucleus of the SOM in 5xFAD. The data are presented as the mean ± S.E.M. **p < 0.01; ****p < 0.001 as determined by the unpaired t-test. Scale bars = 5 μm. **H** Axonal spheroids were observed in the vicinity of septal Aβ plaques for SOM by immunofluorescence staining for SST (white dashed line) in the 5xFAD; Thy1-YFP mouse septum. **I** Immunofluorescence analysis of the septum area in 5xFAD; Thy1-YFP mice showed that the axonal spheroids of SOM are greatly induced in the vicinity of Aβ plaques (Amylo-Glo) as early as 2-months old when Thy1-YFP-positive projection neurons initiate their axonal dystrophy. Scale bars = 5 μm. **J** Images of SOM in WT and 5xFAD mice were immunostained for Hoechst, CD63, ACIII, and SST. CD63 aggregates formed in the brightly stained perinuclear SST and primary cilia were short and weakly labeled in the SOMs of 5xFAD mice. Scale bar = 2 μm

The 5,468 neurons characterized by the high expression of *Meg3* and *Atp1b1*, were further examined in our single-cell dataset (Fig. 6A, Additional file 1: Fig. 8C). We introduced independent component analysis into the dimensional reduction to decompose the gene expression matrix into 127 well-known hippocampal marker genes as independent components and found 9 distinct neuronal subclusters using canonical marker analysis (Fig. 6B, Additional file 15: Table 7). We examined the neuronal transcriptome response to the production of Aβ. The proportion of neurons with somatostatin (*Sst*), chromogranin B (*Chgb*), and calbindin (*Calb2*) markers was significantly decreased in 5xFAD mice (Fig. 6C) casting them as vulnerable neuronal types. To further understand the three neuron types, we conducted differentially expressed genes analysis between 5xFAD mice and age-matched WT control mice, revealing 620 upregulated genes (log_2_Fold Change > 0.25, *p*-value < 0.01) and 234 down-regulated genes (log_2_Fold Change < -0.25, *p*-value < 0.01) in SOM, 738 upregulated genes (log_2_Fold Change > 0.25, *p*-value < 0.01) and 186 down-regulated genes (log_2_Fold Change < − 0.25, *p*-value < 0.01) in Chgb-expressing neurons, and 816 upregulated genes (log_2_Fold Change > 0.25, *p*-value < 0.01) and 185 down-regulated genes (log_2_Fold Change < − 0.25, *p*-value < 0.01) in Calb2-expressing neurons (Fig. 6D, Additional file 15: Table 7). A closer examination of the differentially expressed genes and gene-set enrichment analysis demonstrated that three subclusters shared common pathways, which included secretion, secretion by cell, and regulation of secretion by cell, within the GO biological process signatures (Fig. 6E). Remarkably, two distinct subtypes of neurons, characterized by the presence of *Sst* and *Calb2*, were affected by Aβ expression, although they did not produce Aβ via Thy1-promoter-driven transgenes in 5xFAD mice. As the *Thy1* gene was expressed in neurons with the *Chgb* marker (Fig. 6B), we compared the gene-set enrichment analysis of *Chgb*-expressing neurons (human transgenic APP-expressing neurons driven by Thy1-promoter) to that of SST-expressing neurons (SOM, neurons lacking human transgenic APP expression due to the lack of Thy1-promoter activity) (Fig. 6E). Notably, both Chgb- and SOM showed reduced expression of the “non-motile ciliary assembly” pathway linked to the “secretion” or “secretion by cell” GO biological process pathways via Dynll1, a member of the dynein light chain family of proteins (Fig. 6F). Both neuronal types showed similar dampening of pathways regardless of their differences in Thy1-promoter-driven transgene expression, except for the upregulation of the regulation of phagocytosis. These results may suggest that axonal dystrophy preferentially occurs through the accumulation of secretory vesicles and deficits in the downstream signaling cascades of the primary cilia, which do not depend on APP production.

To confirm our finding that SOM in the hippocampus were selectively prone to extracellular Aβ, we used 5xFAD; Thy1-YFP mice, and WT control mice to stain SOM in the hippocampus. The density of SOM in the CA1 and the dentate gyrus hilus was diminished in 5xFAD mice (Fig. 6G). Unlike the Sst expression in the SOM of WT mice, the SOM in 5xFAD mice was densely packed in the perinucleus (white dashed line, Fig. 6G). We revisited the axonal spheroids in the septum area to examine the Sst-positive DNs (Fig. 6H). Our results showed that as early as 2 months in 5xFAD mice, Sst-expressing neuronal axons, which are negative for YFP signals, exhibited prominent spheroids with Thy1-YFP-positive axonal spheroids surrounding the forming Aβ plaques (Fig. 6I).

Furthermore, our immunocytochemical analysis of SOM in the hippocampus revealed that CD63-positive puncta were localized in the perinucleus of SOM in 5xFAD mice. Interestingly, perinuclear aggregation of SST in 5xFAD mice may reflect defective axonal transport of vesicles containing SST. (Fig. 6J). In addition, we observed a decrease in the signal of the primary ciliary marker (ACIII) in the SOM of 5xFAD mice compared to WT control mice, supporting our above single-cell RNA-sequencing analysis. Our findings indicate that hippocampal SOM might be selectively vulnerable to extracellular Aβ via the impairment of vesicle exocytosis and primary cilia function.

## Discussion

This study found that SOM in the hippocampus connecting the septum were vulnerable to extracellular Aβ plaques. Studies have shown that RORB-expressing excitatory neurons in the EC [45] and the frontal cortex [56], as well as SST-positive interneurons in the frontal cortex [8], are more prone to neuronal susceptibility than other parts of the brain. Dysfunctional SOMs have been identified as a hallmark of AD, and loss of SOMs in the cortex is associated with AD pathologies [10, 15, 65]. Previous research has shown a decrease in SST expression in the cortex and hippocampus of AD patients. In addition, SST administration has been shown to be effective in improving cognitive impairment in AD patients [12, 15]. Recently, it has been shown that SOMs are more vulnerable than other inhibitory neurons, such as parvalbumin neurons, in TgF344-AD rats [49]. When Aβ was injected into the hippocampus, it was reported to have a deleterious effect on the viability of SOM to connect the septum but had little effect on parvalbumin-expressing neurons [74]. Similarly, our study showed that *Slc32a1*-expressing interneurons, including parvalbumin-expressing neurons, were less susceptible to Aβ plaques than SOM. Because our scRNAseq data were obtained from the mouse hippocampus at 3, 5, and 6 months of age, we thought that the major pathological changes in the temporal lobe, including the medial septum, hippocampus, and EC, might be initiated with axonal dystrophy of the hippocampal SOM to send their axons to the septum. This initial stage is not easily detectable, and massive synaptic loss and subsequent functional deficits have been noted. In hippocampo-septal projecting neurons, the efficacy of transporting proteins from the axon terminals to the perinuclear lysosomes could be hampered by the extensive distances involved. The already declining lysosomal clearance activity of neurons could be exacerbated by the accumulation of damaged organelles and aggregated proteins. Thus, the subclinical conditions of primary cilia defects may surface as a risk for vulnerable neurons. Many studies are needed to determine whether SST-expressing axonal dystrophy exacerbates the neurite dystrophy of hippocampal glutamatergic neurons or whether interfering with SST-expressing neuronal loss could delay the progression of massive hippocampal neuronal degeneration. However, this cellular pathology explains the early cognitive symptoms of AD, such as memory impairment and spatial disorientation [11]. Since the hippocampo-septal system is interconnected by the fimbria-fornix complex, loss of inhibitory hippocampo-septal connectivity may cause deficits in oscillatory activity such as theta rhythm and coordination of learning and exploratory behavior.

SST-expressing double projecting interneurons within the hippocampus and the hilus of the dentate gyrus possess a retrohippocampal axon that targets synaptic sites in the CA3, DG, and a long-range axon connecting to the septum [5]. Among the double projecting interneurons, SOM bearing a hippocampo-septal projecting axon may comprise vulnerable neurons such as entorhinal RORB-expressing excitatory neurons in the AD brain [31, 45]. Notably, our findings suggest that SOM, which do not produce APP, are susceptible to extracellular Aβ, and their transcriptome signatures showed similarity with a specific group of neurons that express *Thy1* and produce Aβ under the control of the *Thy1* promoter. Our results further revealed that Aβ plaques had a significant impact on the downregulation of pathways related to cell secretion and cilium assembly in both neuronal types. Collectively, the axonal dystrophy of SOM was manifested by the accumulation of EVs regardless of Aβ encapsulation, and the dysfunction of primary cilia was associated with this pathology.

One possible explanation is that axonal spheroids, composed of accumulated vesicles including EVs, could be affected by nearby toxic Aβ that damages presynaptic membranes required for EV secretion. This concept is supported by the fact that a certain population of SST-positive neurons in the hippocampus send long-distance axons to the septum [24]. Axonal transport of various vesicles, including neuropeptide-positive dense core vesicles and endosome-lysosomal vesicles, is critical for the homeostasis of presynaptic function. Aβ accumulation derived from the axon terminals of *Thy1*-expressing projection neurons could disrupt the axonal transport of SOM. This can lead to dystrophic, vesicle-accumulating axons close to Aβ plaques, which feature accumulated presynaptic materials, implying the failure of synaptic release. The accumulation of CD63-positive EVs and SST-containing vesicles in the perinucleus indicates that certain proteins that are essential for vesicular transport and release may be stuck with SST-containing vesicles near the nucleus in the vulnerable SOM [61, 81], which results as a result of vesicular release at the axon terminus.

In addition, neuronal primary cilia are at the intersection of extracellular and intracellular signaling pathways that control the function of subcellular organelles like the proteasome, autophagosome, and microtubule-rich basal body [6, 57]. These processes may interact with internalized Aβ processed through the MVB or endo-lysosomal pathways [76]. Aβ not only affects intracellular vesicular trafficking but could also affect primary cilia function directly or indirectly via its cognate binding receptors such as LRP1 [15, 50, 75]. Thus, defective primary cilia could make it more difficult for vesicles to move along axons and exacerbate dystrophic axons in hippocampal neurons as Aβ spreads. These findings support the idea that Aβ plaques may disrupt cellular processes related to neuronal primary cilia and EV trafficking in neurons, leading to the formation of axonal spheroids and synaptic damage. Further investigation into the mechanisms underlying the interaction between Aβ, dystrophic axons, and primary cilia is likely to provide novel insights into the development of therapeutic targets for neurons, including SOM, which are more susceptible to the early stage of AD.

Our study revealed that loss of *Ift88* may make hippocampal neuronal axons more vulnerable, which may involve the primary cilia directly or indirectly. The primary cilia are uniquely positioned to limit age-related risk factors for the disease. For example, mutations in the *Pkd1* gene are known to cause cysts to slowly form in the kidney [41]. This shows that even if the cilia aren't working properly, other things are needed for the tissue to break down. With advancing age, primary cilia may accumulate risk factors that can make pathological problems in cells worse. In addition, primary cilia and neuronal axons together have acetylated tubulin, which is important for keeping their structures intact. Thus, axonal dystrophy resulting from *Ift88* inhibition may involve microtubule-associated proteins, including post-translational modifications of tau and motor proteins for protein transport. The centrosome is the main microtubule organizing center, which establishes axon elongation and cilia formation and mediates the transport of axonal proteins. Restricted expression of acetylated tubulin near the centrosome defines a stable location for axon growth [77]. The mother centriole synthesizes and releases acetylated microtubules for transport down the axon and the primary cilia, and modulation of primary cilia signaling such as AKT and AC3 controls axonal behavior [1, 26]. The acetylation modification of tubulin is preferable for the primary cilia [78] and the attachment of the primary cilia with a centriole tightens the regulation of the transport of acetylated tubulin into the primary cilia.

Structural or functional alterations in the primary cilium are observed in AD, resulting in a significantly shorter length of primary cilia compared to controls [37, 47]. It has been reported that primary cilia bearing the SSTR3 receptor were shorter in the hippocampus of 3xAD mice than in WT mice [9]. Additionally, the length pattern of primary cilia was altered in the enteric neurons of 5xFAD mice [52]. Finally, cilia morphology in the hippocampus of APP/PS1 mice was altered [28]. Ciliary dysfunction associated with Aβ has been reported to reduce the connectivity of neuronal circuits and cause synapse loss [25, 75]. Another possible explanation for the observed ciliary dysfunction and formation of axonal spheroids resulting from the Aβ burden is the role of primary cilia in the cell cycle. Primary cilia, which make use of mitotic spindle-like molecular machinery, may influence the regulation of the cell cycle machinery in neurons [6, 57]. In normal physiological conditions, neurons don’t usually experience replication stress. However, the extracellular environment may trigger cell cycle progression, thereby affecting the preventative function of primary cilia [62]. The neuroinflammatory microenvironment of AD may change the state of the cell cycle of non-neuronal cells through the release of cytokines and chemokines. The cell cycle checkpoint of neuronal primary cilia may be activated by cues inducing cell cycle re-entry under pathological and neuroinflammatory conditions. As neurons leave the G0 state, their ability to maintain optimal protein synthesis and translocation for post-mitotic functional synapses may be hampered, making the axon vulnerable. These questions, however, remain to be addressed.

Furthermore, the function of primary cilia may be disrupted, which could affect how acetylated tubulin and motor and stabilizing proteins like tau move into the axon compartment. Microtubules are important integral components of cilia and axons, and tau proteins help them stay in place. It is well known that hyperphosphorylation of tau proteins leads to their accumulation into neurofibrillary tangles, which can potentially destabilize microtubules [27, 56]. The dysfunction of primary cilia may be due to the accumulation of hyperphosphorylated tau proteins following neuronal exposure to Aβ. The aggregation and fibrillation of microtubule-stabilizing proteins such as tau may have potential negative effects on the structure and function of primary cilia. Further research is required to understand how primary cilia control microtubules and tau proteins in post-mitotic neurons throughout aging and pathological circumstances like AD.

## Limitations

Our study presented evidence that the tetraspanin membrane proteins [48], which are implicated in EV formation and secretion as well as MVB trafficking, are one of the major proteins that interact with Aβ. This suggests that Aβ, whether intracellularly expressed or taken up by neurons and delivered to early endosomes, is transported by vesicles, and accumulates at axon terminals. Immunocytochemical analysis of both in vitro and in vivo samples revealed that the CD63 protein, a highly abundant protein in EVs and present on the membranes of the MVB, was localized at axon terminals, forming axonal spheroids. Together, our results demonstrate that exosome-like vesicles are positioned as the central mediator of axonal spheroid formation, with a mixture of vesicles associated with the presynapse [54, 83] in the AD brain.

While there has been recent research on outside-in signaling in the development of DNs [54], we believe this is the first study of inside-out vesicles affecting DNs in AD. It is widely assumed that Aβ is sorted into MVBs and sent to the lysosome for degradation. Axonal spheroids are also known to contain the abnormal accumulation of organelles and vesicles in several degenerative diseases [2, 22, 36] and their enlargement is caused by the accumulation of aberrantly enlarged LAMP1-positive endo-lysosomal vesicles [83]. Our interactome analysis of CD63 proteins in synapses provided clues that several proteins deficient in mouse models of AD were involved in the endosomal-lysosomal machinery in addition to vesicle exocytosis. We did not further elucidate how endo-lysosomal deficits affect the accumulation of EVs; in particular, we did not clarify the mechanisms that explain how primary cilia were involved. Future studies are needed to fully characterize the vesicular landscapes in the dystrophic synapses in AD.

## Conclusion

In conclusion, the purpose of this investigation was to determine how Aβ burden affects the presynaptic terminals of specific neurons in the hippocampo-septal tract and the molecular mechanisms that lead to synapse loss and network dysfunction. Our study established the presence of axonal spheroids adjacent to Aβ plaques and their deleterious effect on synapse loss and network dysfunction during AD progression. Our study also revealed a deficit in signaling pathways related to primary cilia and vesicle release, leading to the accumulation of secretory EVs at the presynaptic terminals. Furthermore, our study found that SOM in the hippocampo-septal tract were selectively vulnerable to Aβ and exhibited the previously described features associated with axonal spheroid formation. These findings shed light on the molecular and cellular mechanisms underlying the initial loss of synapses in AD pathogenesis.

### Supplementary Information

**Additional file 1**. Supplementary figures 1–8

**Additional file 2**. Supplementary Video 1 (A): Three-dimensional visualization of the YFP-fluorescence from Thy1-YFP

**Additional file 3**. Supplementary Video 1 (B): Three-dimensional visualization of the YFP-fluorescence from 5xFAD; Thy1-YFP mice

**Additional file 4**. Supplementary Video 2. Three-dimensional visualization of axon terminals adjacent to Aβ plaques in the septum of the 5xFAD; Thy1-YFP mice

**Additional file 5**. Supplementary Video 3 (A): Three-dimensional visualization of anterograde tracing using AAV-Cre virus into the dentate gyrus of Ai6 mice

**Additional file 6**. Supplementary Video 3 (B): Three-dimensional visualization of anterograde tracing using AAV-Cre virus into the dentate gyrus of 5xFAD; Ai6 (B) transgenic mice

**Additional file 7**. Supplementary Table 1. Differentially expressed proteins between proteomic profiles of synaptosomes obtained from 5xFAD (12-month-old, n = 4, male) and WT (12-month-old, n = 4, male) control mouse brains and gene-set enrichment analysis utilizing the biological process in Gene Ontology

**Additional file 8**. Supplementary Table 2. Differentially expressed proteins between proteomic profiles of lysates obtained from WT (2-month-old, n = 4, male) and 5xFAD mice (2-month-old, n = 4, two female and two male mice) mouse brain and gene-set enrichment analysis utilizing the biological process in Gene Ontology

**Additional file 9**. Supplementary Table 3. Differentially expressed proteins between Aβ1-42-treated and vehicletreated neurons and gene-set enrichment analysis utilizing the biological process in Gene Ontology (n = 3 per group)

**Additional file 10**. Supplementary Table 4. Differentially expressed proteins between APP-immunoprecipitated synaptic proteins of 5xFAD (6 months old, n = 4, one female and three male mice) and WT (6 months old, n = 4, two female and two male mice) control mouse brain and gene-set enrichment analysis utilizing the biological process in Gene Ontology

**Additional file 11**. Supplementary Video 4. Three-dimensional visualization of CD63-EGFP expression after introduction of an AAV1 vector expressing CD63-EGFP into the vDG of WT control mice

**Additional file 12**. Supplementary Video 4. Three-dimensional visualization of CD63-EGFP expression after introduction of an AAV1 vector expressing CD63-EGFP into the vDG of 5xFAD mice

**Additional file 13**. Supplementary Table 5. Differentially expressed proteins between CD63-immunoprecipitated synaptic proteins of 5xFAD (6 months old, n = 10, four female and six male mice) and WT (6 months old, n =11, one female and ten male mice) control mouse brain and gene-set enrichment analysis utilizing the biological process in Gene Ontology

**Additional file 14**. Supplementary Table 6. Differentially expressed proteins between siIft88-transfected and siContransfected neurons upon Aβ1-42 treatment and gene-set enrichment analysis utilizing the biological process in Gene Ontology (n = 3 per group)

**Additional file 15**. Supplementary Table 7. Representative marker expression of 10 distinct neuronal subclusters, differentially expressed genes of Sst, Chgb, and Calb2-expressing neurons, and their gene-set enrichment analysis utilizing the biological process in Gene Ontology

## Data Availability

The proteomics data have been deposited with the ProteomeXchange Consortium via the PRIDE partner repository (identifier PXD041709) and scRNAseq data can be accessible via the GEO repository (GSE230240). All materials are available by contacting the corresponding author.

## Abbreviations

Aβ: Amyloid-β
DNs: Dystrophic neurites
AD: Alzheimer’s disease
APP: Aβ precursor protein
EC: Entorhinal cortex
Ift88: Intraflagellar transport protein 88
pSer8 Aβ: Aβ with phosphorylation at serine 8
LRP1: Lipoprotein receptor-related protein 1
SST: Somatostatin
SOM: SST-expressing neurons
Chgb: Chromogranin B
Calb2: Calbindin
TBS: Tris-buffered saline
PBS: Phosphate buffered saline
SDS: Sodium dodecyl sulfate
IP: Immunoprecipitation
co-IP: Co-immunoprecipitation
LC–MS/MS: Mass spectrometry combined with liquid chromatography
PD: Proteome discoverer
PDMS: Polydimethylsiloxane
UMIFM: Unique molecular identifiers filtered mapped
UMAP: Uniform manifold approximation and projection
S.E.M: Standard error of the mean
vDG: Ventral dentate gyrus
scRNAseq: Single-cell RNA sequencing
MVB: Multivesicular body
EVs: Extracellular vesicles
WT: Wild-type
