# Supplementary material for: Abnormal accumulation of extracellular vesicles in hippocampal dystrophic axons and regulation by the primary cilia in Alzheimer’s disease
Source: Acta Neuropathol Commun. 2023 Sep 4;11:142. doi: 10.1186/s40478-023-01637-3 (PMC10478284; doi:10.1186/s40478-023-01637-3)
Supplement: Supplementary file 1 — Additional file 1. Supplementary figures 1–8 [file 40478_2023_1637_MOESM1_ESM.docx]

**Abnormal accumulation of extracellular vesicles in hippocampal dystrophic axons and regulation by the primary cilia in Alzheimer’s disease**

Abbreviated title: Primary cilia defects and axonal vulnerability of Alzheimer’s disease

**Jaemyung Jang, Seungeun Yeo, Soonbong Baek, Hyun Jin Jung, Mi Suk Lee, Seung Hee Choi, Youngshik Choe^*^**

**
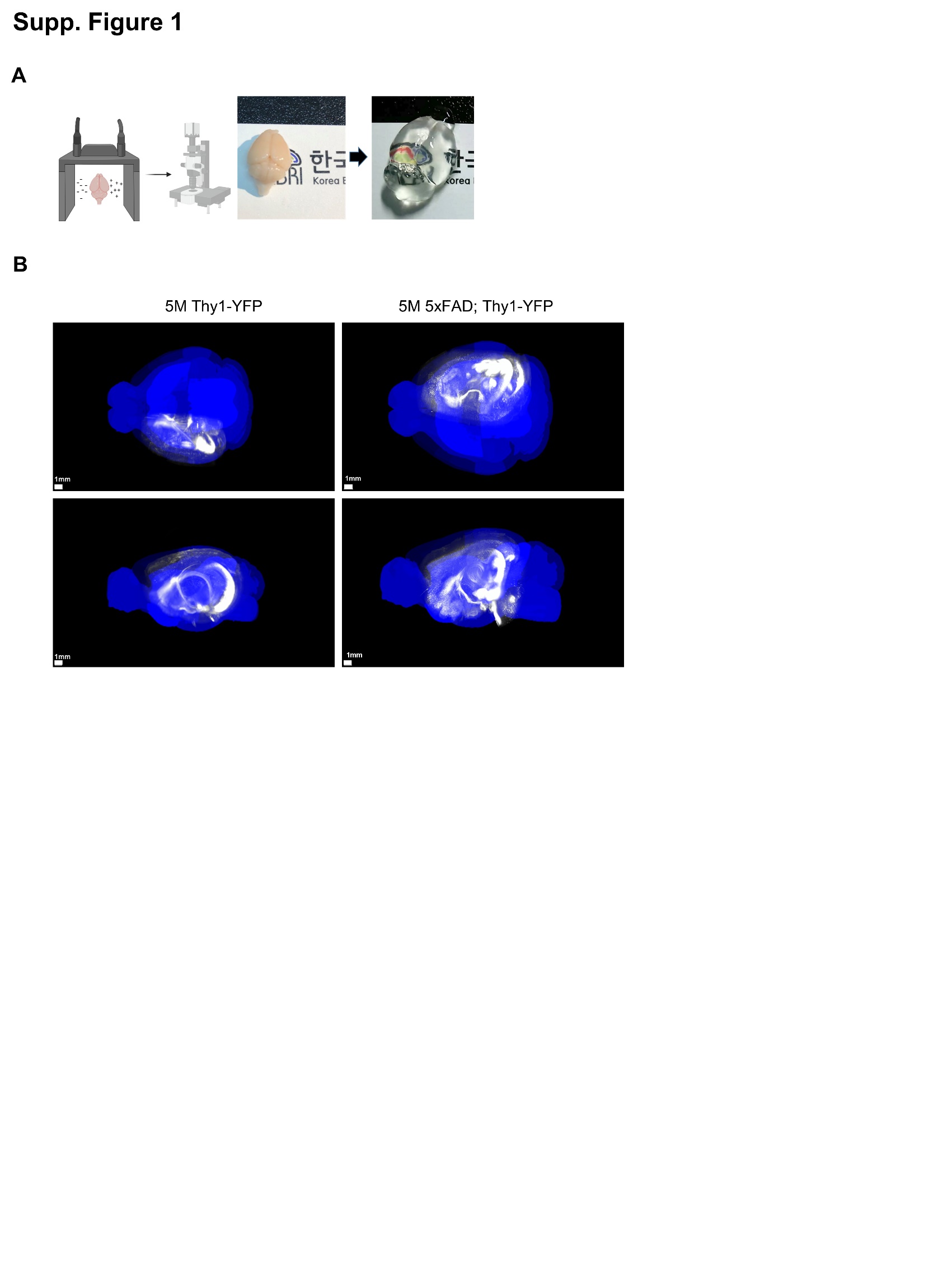
**

Supp. Fig. 1. Whole-brain imaging of 5xFAD; Thy1-YFP transgenic mice

1. Schematic and example of whole-brain imaging using the active clarity technique.
2. Dorsal and lateral views focusing on the hippocampo-septal pathways visualized with the Thy1-YFP reporter in WT and 5xFAD mice at five months of age (n=4, female). Scale bars = 1mm.


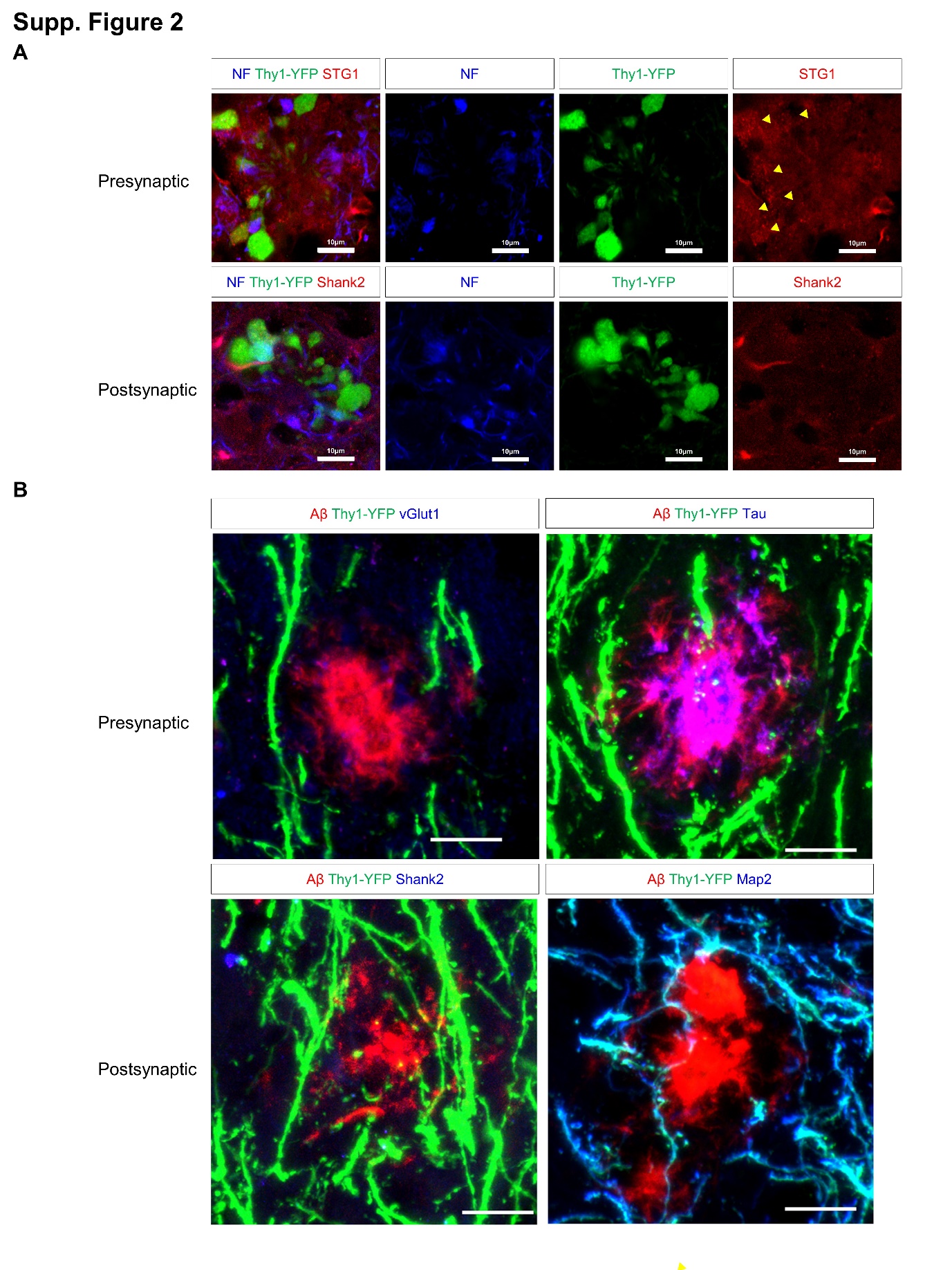


Supp. Fig. 2. Representative images of axonal spheroids in the vicinity of Aβ plaque

1. Swollen dystrophic neurites of 5xFAD; Thy1-YFP mice at six months of age were immunostained for synaptotagmin-1 (STG1), and Shank2 to co-stain pre and postsynaptic compartments, respectively. Neurofilament (NF) was used to label dystrophic neurites. Representative images of four independent experiments were presented (n=4, female). Scale bars = 10μm,
2. Lack of swollen dystrophic neurites in the molecular layers of the dentate gyrus rich in YFP-positive dendrites of dentate granule neurons at seven months of age of 5xFAD; Thy1-YFP mice. Sections were immunostained for Aβ, and presynaptic markers such as vesicular glutamate transporter 1 (vGlut1), and Tau, and postsynaptic markers such as Shank2, and Map2. Representative images of four independent experiments were presented (n=4, female). Scale bar = 5μm,


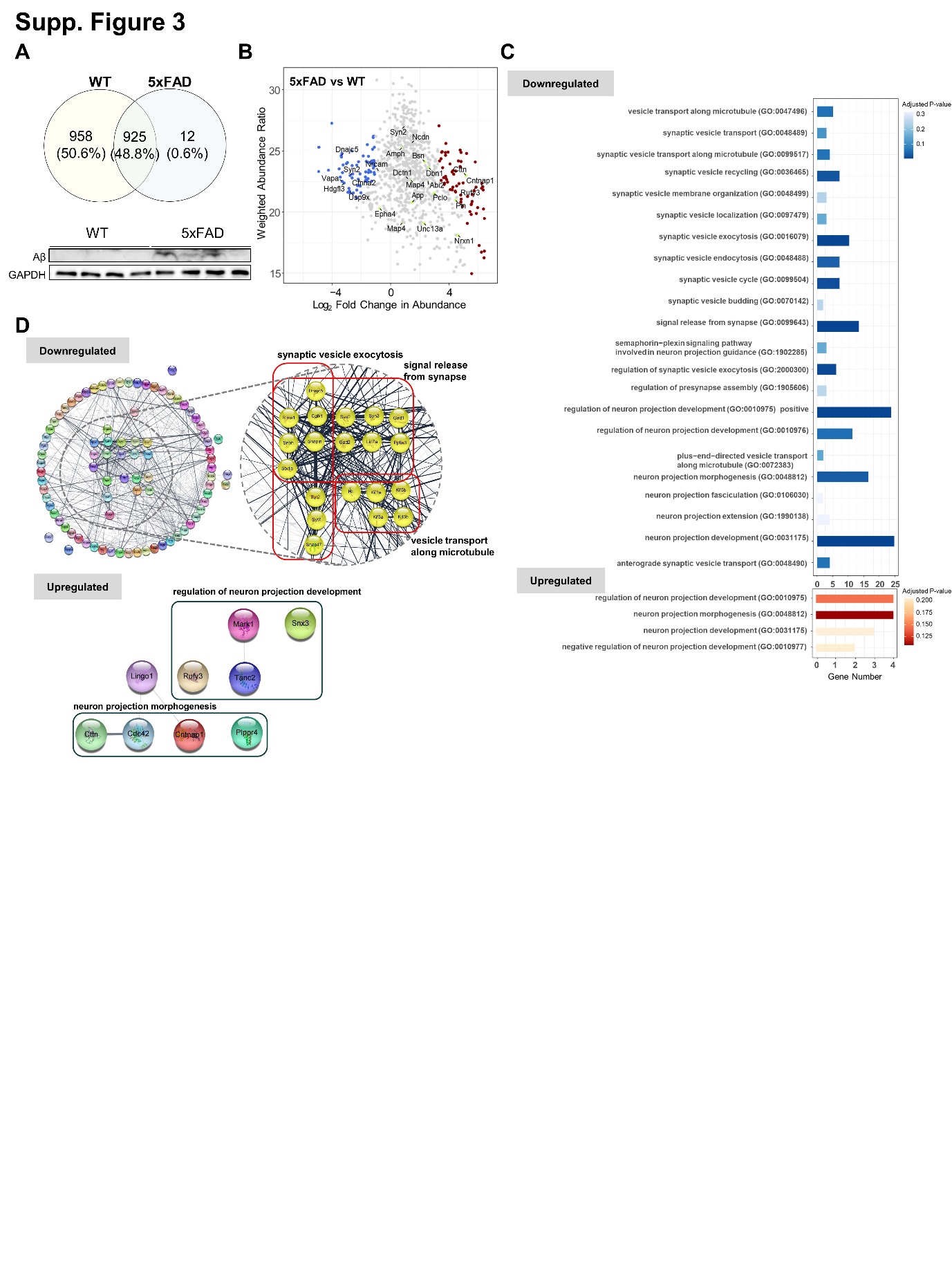


Supp. Fig. 3. Representative images of axonal spheroids in the vicinity of Aβ plaque

1. Lysates of the hippocampo-septal tracts of WT (2-month-old, n = 4, male) and 5xFAD mice (2-month-old, n = 4, two female and two male mice) were analyzed by LC-MS/MS to investigate the biological processes underlying the initial stage of DN formation. Western blot analysis showed slight expression of Aβ in 5xFAD at 2 months old.
2. A scatter plot shows 105 up-regulated (log_2_ fold-change > 0.25, adjusted p-value < 0.1) and 1,023 down-regulated proteins (log2 fold-change < -0.25, adjusted p-value < 0.1) as a result of the differentially expressed protein analysis. Shown are the fold changes in protein abundance between 5xFAD (2-month-old, n = 4, two female and two male mice) and WT (2-month-old, n = 4, male) hippocampo-septal tissues and the weight value of this quantification. The position of the representative proteins selected for gene-set enrichment analysis is indicated in colors (blue=downregulated, red=upregulated). Proteins with green dots represent the expression of representative proteins upregulated in 12-month-old 5xFAD synaptosomes as shown in Figure 2G.
3. The results of the gene set enrichment analysis based on GO biological processes were retrieved using the same keywords as in Figure 2F and plotted as two bar graphs.
4. PPI network analysis using the STRING database identified two different networks with a network of 88 downregulated proteins with 518 connections and a network of 9 upregulated proteins with 4 connections.


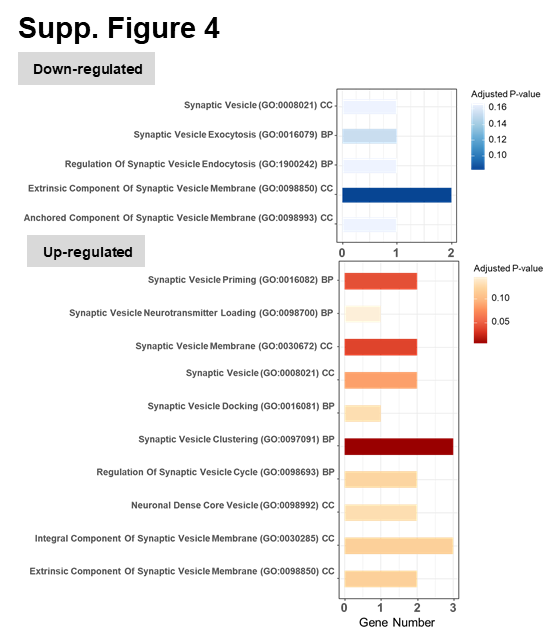


Supp. Fig. 4. The Synaptic Gene Ontologies annotated the genes present in the synaptosomes that were immunoprecipitated by antibodies for APP C-terminals from both 5xFAD and WT control samples. The results show that the proteins interacting with Aβ are related to synaptic vesicles including dense core vesicles present in synapses.


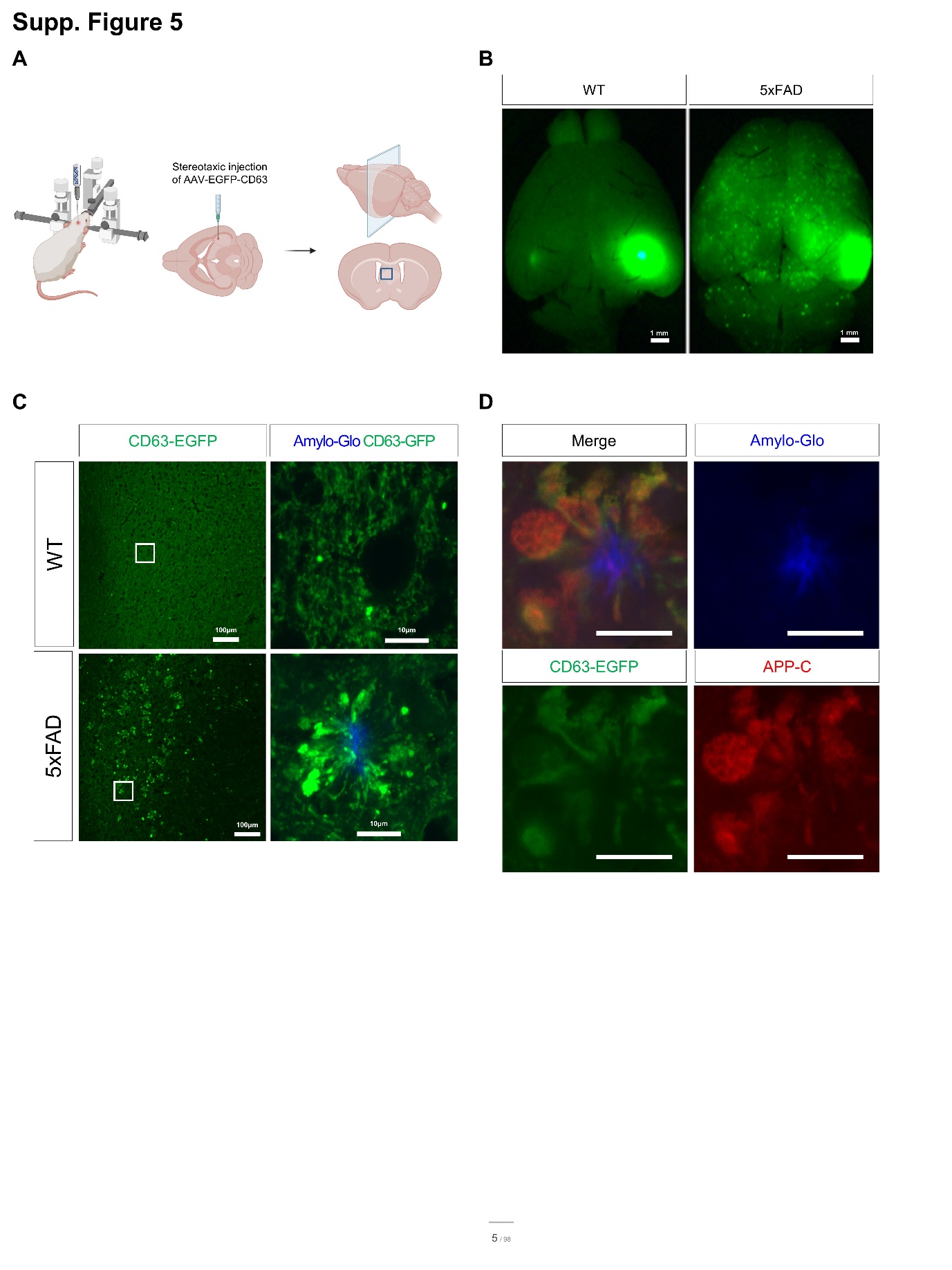


Supp. Fig. 5. Accumulation of CD63-EGFP vesicles in the axonal spheroids of the 5xFAD mouse brain

1. Workflow for injection of AAV vector expressing CD63-EGFP into the vDG and brain-wide visualization of CD63-EGFP
2. A representative dorsal view of CD63-EGFP expression in the WT control and 5xFAD mouse brain (n=4, female). Scale bars = 1mm
3. Coronal sections of CD63-EGFP-injected brains at the level of the septum. Higher magnification images were presented from the white box area. Amylo-Glo was used to counter-stain the Aβ plaques (blue) (n=4, female). CD63-EGFP_ Scale bar = 100μm, Amylo-Glo/ CD63-EGFP_Scale bar = 10μm
4. A representative fluorescence image of axon terminals proximal to Aβ, immunostained for Amylo-Glo (blue), C-terminal fragments of APP (APP-C, red), and CD63-EGFP (green). Representative images of four independent experiments were presented (n=4, female). Scale bars = 10μm


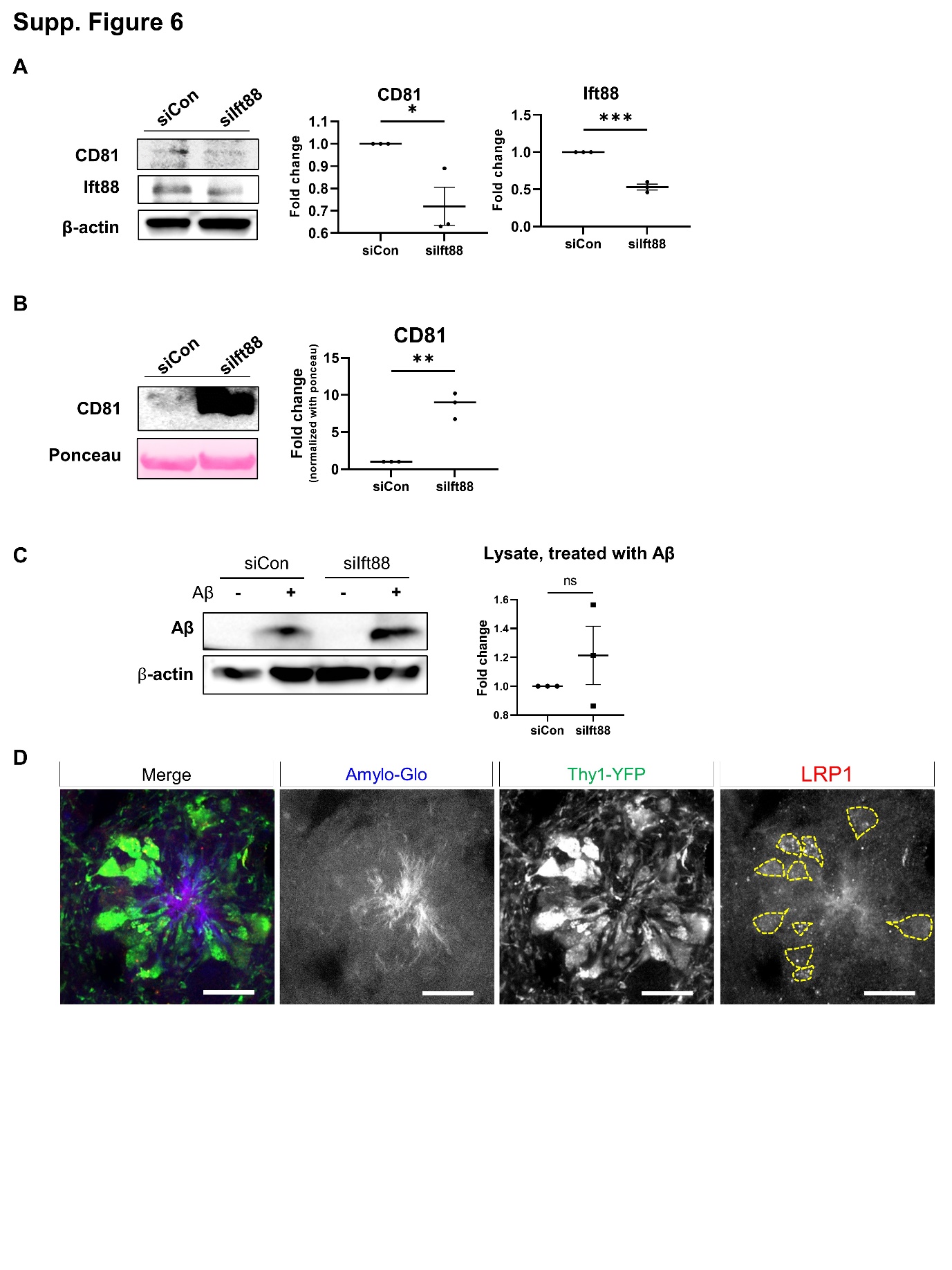


Supp. Fig. 6. Facilitation of EV secretion by conditional knock-down of *Ift88* expression in the primary neurons

1. Western blot analysis using whole-cell lysates obtained from primary neurons after siRNA (40nM) transfection showed a significant reduction in CD81. The data of the three independent experiments (n = 3 per group) were analyzed by the unpaired t-test. *, p < 0.05; ***, p < 0.001
2. Western blot analysis using EVs collected from the culture medium of primary neurons after siRNA (40nM) transfection showed a significant increase in CD81. The data of the three independent experiments (n = 3 per group) were analyzed by the unpaired t-test. **, p<0.01
3. Western blot analysis using whole-cell lysates from primary neurons after siRNA (40nM) transfection and 1μM Aβ treatment showed that Aβ uptake was variable after siIft88 transfection. The data of the three independent experiments (n = 3 per group) were analyzed by the unpaired t-test (ns; non-significant).
4. Expression of Lrp1, a receptor for Aβ, in the dystrophic neurites. The septum of 5xFAD mice expressing a Thy1-YFP reporter was used to stain dystrophic neurites (YFP) and Lrp1. Amylo-Glo was used to counterstain the Aβ plaques. Dystrophic neurites with punctuated Lrp1 expression were denoted by dashed lines (n=4, female). Scale bars = 10μm


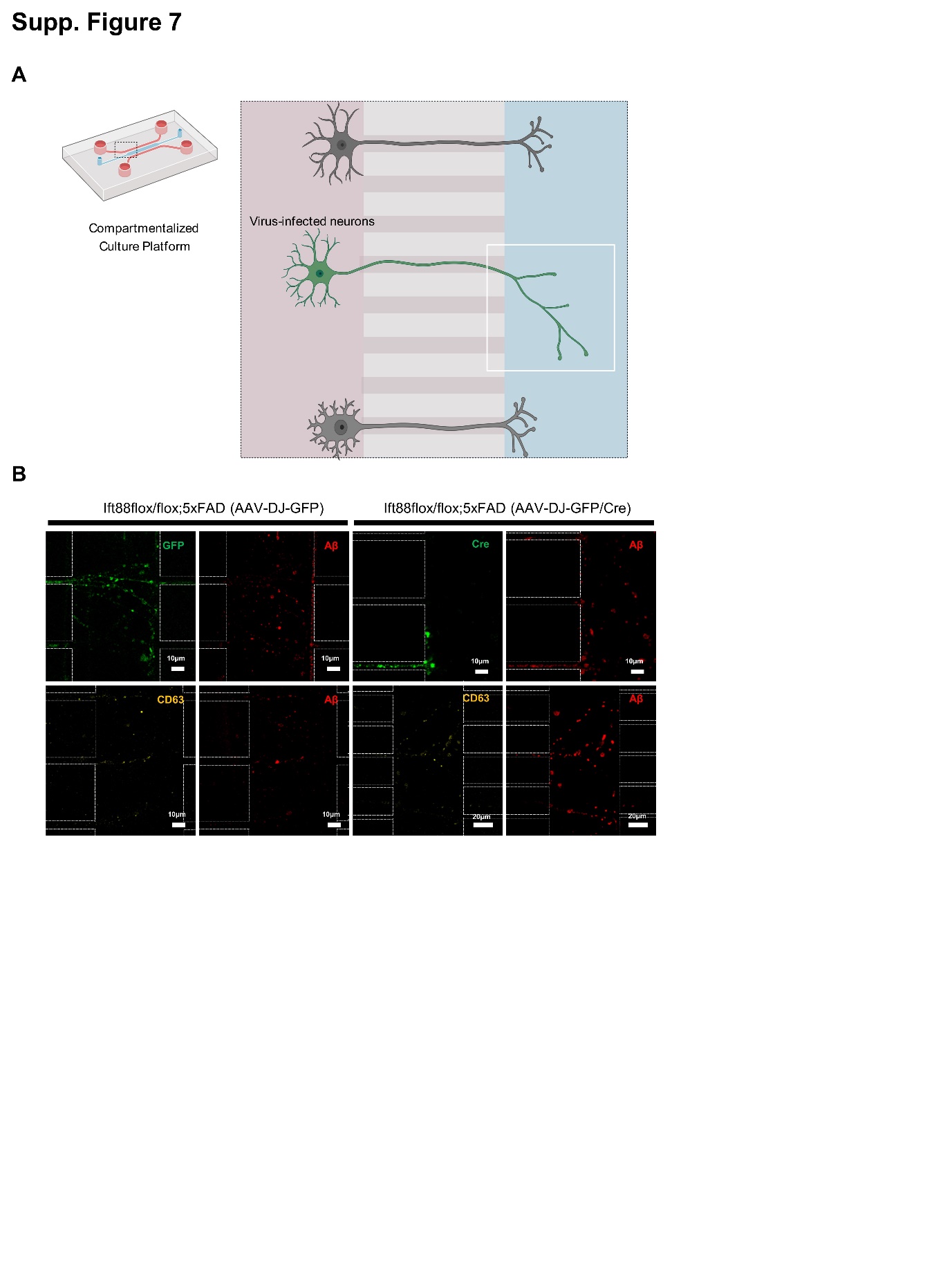


Supp. Fig. 7. The accumulation of Aβ-containing EVs by disruption of primary cilia through *Ift88* knock-down

1. Schematic of the compartmentalized neuron culture platform. Primary hippocampal neurons were established from 5xFAD mice carrying floxed *Ift88* genes (5xFAD; Ift88flox/flox) to induce Aβ expression in the neurons. Neurons were plated in the left microchannel (red). Axons passed through microgrooves and diverged in the middle microchannels (blue). Further analysis was performed on the white box in the schematics.
2. Using hippocampal neurons grown in compartmentalized neuron culture platform with Cre/LoxP-mediated *Ift88* deletion, the area of axon terminals containing intraneuronal Aβ deposition was significantly increased. The co-localization coefficients of CD63 and Aβ were significantly increased in primary neurons with *Ift88* knock-down. Scale bars = 10μm for Cre-infected samples (n = 3) and 20μm for GFP-infected samples (n = 3)


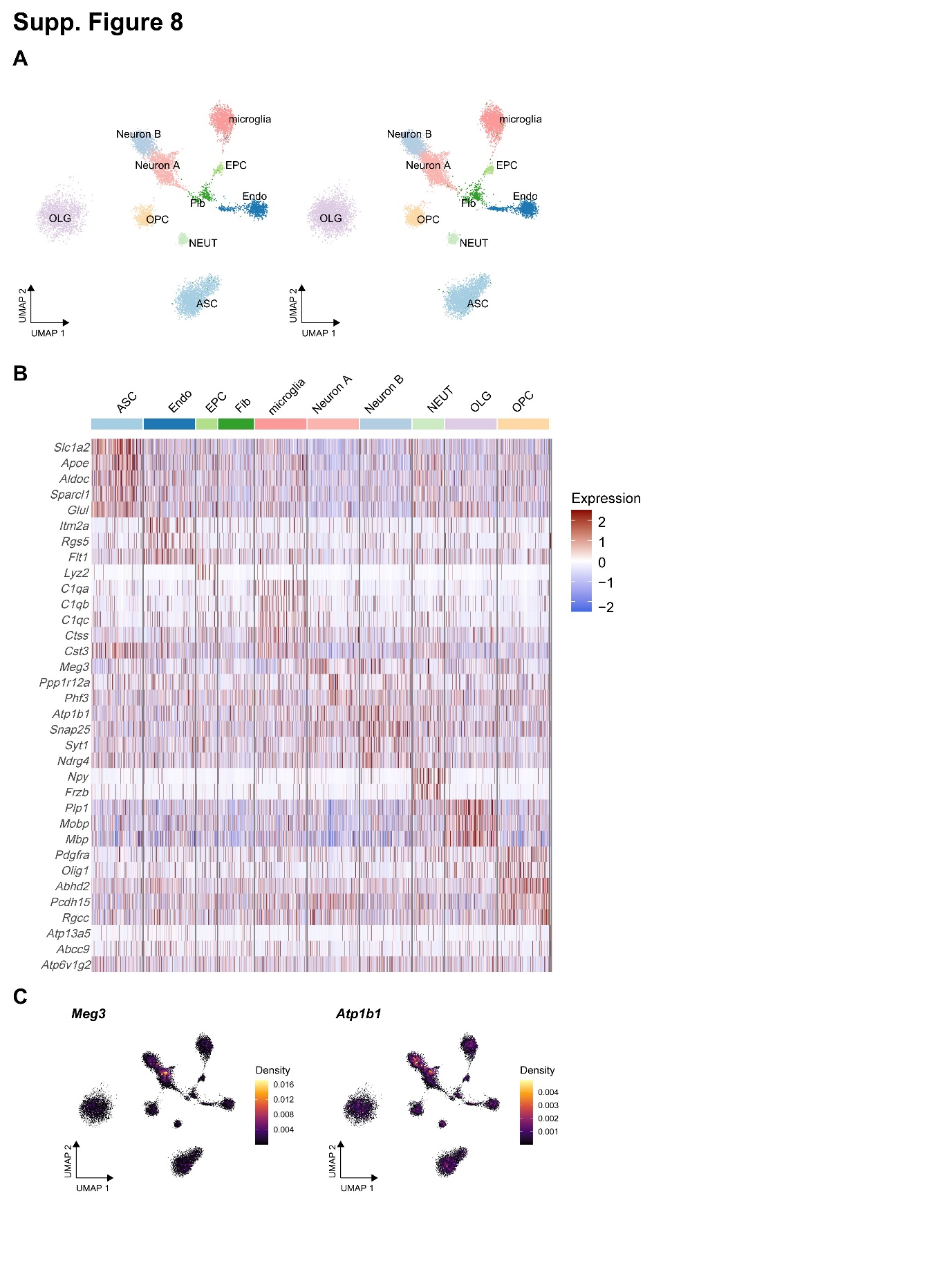
Supp. Fig. 8. Single cell transcriptome profiling of the hippocampal tissues of WT (3 months old, n = 3, male mice; 6 months old, n =1 female mouse) and 5xFAD mice (3 months old, n = 4, male mice; 6 months old, n =2, female mice).

1. Reduced dimensionality (UMAP) visualization depicting the overall tissue distribution of the major cell types of the hippocampi of WT and 5xFAD mice (ASC: astrocyte, Endo: endothelial cells, EPC: ependymal cells, Fib: Fibroblast, NEUT: neutrophils, OPC: oligodendrocyte precursor cells, OLG: oligodendrocytes)
2. Heatmap indicating the expression of marker genes in the indicated cell types
3. UMAP plot showing the subtypes of neurons derived from WT and 5xFAD mouse hippocampal tissues. Each cluster is color-coded according to cell type.

**Supplementary Information**

Supplementary Table 1. Differentially expressed proteins between proteomic profiles of synaptosomes obtained from 5xFAD (12-month-old, n = 4, male) and WT (12-month-old, n = 4, male) control mouse brain and gene-set enrichment analysis utilizing the biological process in Gene Ontology

Supplementary Table 2. Differentially expressed proteins between proteomic profiles of lysates obtained from WT (2-month-old, n = 4, male) and 5xFAD mice (2-month-old, n = 4, two female and two male mice) mouse brain and gene-set enrichment analysis utilizing the biological process in Gene Ontology

Supplementary Table 3. Differentially expressed proteins between Aβ1-42-treated and vehicle-treated neurons and gene-set enrichment analysis utilizing the biological process in Gene Ontology (n =3 per group)

Supplementary Table 4. Differentially expressed proteins between APP-immunoprecipitated synaptic proteins of 5xFAD (6 months old, n = 4, one female and three male mice) and WT (6 months old, n = 4, two female and two male mice) control mouse brain and gene-set enrichment analysis utilizing the biological process in Gene Ontology

Supplementary Table 5. Differentially expressed proteins between CD63-immunoprecipitated synaptic proteins of 5xFAD (6 months old, n = 10, four female and six male mice) and WT (6 months old, n =11, one female and ten male mice) control mouse brain and gene-set enrichment analysis utilizing the biological process in Gene Ontology

Supplementary Table 6. Differentially expressed proteins between siIft88-transfected and siCon-transfected neurons upon Aβ1-42 treatment and gene-set enrichment analysis utilizing the biological process in Gene Ontology (n = 3 per group)

Supplementary Table 7. Representative marker expression of 10 distinct neuronal subclusters, differentially expressed genes of *Sst*, *Chgb*, and *Calb2*-expressing neurons, and their gene-set enrichment analysis utilizing the biological process in Gene Ontology

Supplementary Video 1. Three-dimensional visualization of the YFP-fluorescence from Thy1-YFP (A) and 5xFAD; Thy1-YFP (B) mice

Supplementary Video 2. Three-dimensional visualization of axon terminals adjacent to Aβ plaques in the septum of the 5xFAD; Thy1-YFP mice

Supplementary Video 3. Three-dimensional visualization of anterograde tracing using AAV-Cre virus into the dentate gyrus of Ai6 (A) and 5xFAD; Ai6 (B) transgenic mice

Supplementary Video 4. Three-dimensional visualization of CD63-EGFP expression after introduction of an AAV1 vector expressing CD63-EGFP into the vDG of WT control and 5xFAD mice
